# Investigating antimicrobial stress responses in *Escherichia coli* induced by high-frequency ultrasound

**DOI:** 10.1016/j.ultsonch.2026.107935

**Published:** 2026-06-27

**Authors:** Irem Soyler, Katie Costello-Gould, Kimon Andreas G. Karatzas, Jorge Gutierrez-Merino, Madeleine Bussemaker

**Affiliations:** a School of Chemistry and Chemical Engineering, University of Surrey, Guildford, United Kingdom; b Fluor Limited, Farnborough, United Kingdom; c School of Chemistry, Food & Pharmacy, Department of Food and Nutritional Sciences, University of Reading, United Kingdom; d School of Veterinary Medicine, Faculty of Health and Medical Sciences, University of Surrey, Guildford, United Kingdom

**Keywords:** High-frequency ultrasound, Bacterial inactivation, Membrane damage, Reactive oxygen species, Epigallocatechingallate (EGCG), Stress adaptation

## Abstract

The antimicrobial mechanisms of high-frequency ultrasound (HFUS) under low-power conditions were investigated using *Escherichia coli* as a model foodborne pathogen, with oxidative and physical contributions evaluated through radical scavenger assays, intracellular reactive oxygen species (ROS) quantification, flow cytometry, and scanning electron microscopy. Bacterial inactivation was strongly frequency-dependent. 760 kHz induced greater intracellular ROS accumulation and membrane permeabilisation than 500 kHz, and radical scavenger experiments confirmed a major ROS contribution to inactivation. The combination of HFUS (760 kHz, 30 W) with epigallocatechin gallate (EGCG; 0.2 mg/mL) produced synergistic antibacterial efficacy, accompanied by enhanced membrane depolarisation, permeabilisation, and ultrastructural damage. Pre-exposure to ultrasound increased bacterial sensitivity to subsequent lethal thermal (56°C) and oxidative (10 mM H_2_O_2_) challenges. Mutant analysis of eight *E. coli* K-12 strains revealed that antioxidant defence systems, particularly glutathione reductase (*gor*), were critical for survival, while deletion of the general stress regulator *rpoS* or oxidoreductase *grxA* increased tolerance, indicating complex interacting stress-response mechanisms. Overall, HFUS inactivation is driven primarily by a combination of chemical and physical mechanisms, encompassing ROS-mediated oxidative damage and cavitation-induced membrane disruption, enhancing bacterial susceptibility to additional antimicrobial stresses, and therefore holds greater potential as a complementary technology than as a stand-alone treatment.

## 1 Introduction

Ultrasound is a non-thermal processing technology that has been applied in the food industry for decades in diverse processes, including homogenisation [1], emulsification, extraction [2], and microbial inactivation [3]. Compared with conventional thermal processing, high-frequency ultrasound (HFUS) can inactivate microorganisms at relatively mild bulk temperatures, helping retain heat-sensitive components and overall product quality [4], [5]. Furthermore, compared with low-frequency ultrasound (LFUS), HFUS is often associated with enhanced sonochemical activity and reactive oxygen species (ROS) generation, providing an additional antimicrobial mechanism based on oxidative stress [6]. These attributes may position HFUS as a promising non-thermal component of hurdle-based preservation strategies [5], [7], [8]. However, broader implementation will require a clearer understanding of the relative contributions of oxidative and physical effects during treatment, as well as the possibility of bacterial stress responses or adaptation under sublethal exposure.

Ultrasound’s antimicrobial potential was first reported in algal microorganisms in the early 1900s [9], and subsequent research has largely attributed these effects to acoustic cavitation [10]. Cavitation involves the formation and collapse of microbubbles, generating highly localised hotspots characterised by extreme temperature and pressure [11]. In addition to these extreme intrabubble conditions, acoustic cavitation generates a non-equilibrium plasma within the collapsing bubbles [12]. This plasma is considered central to modern sonochemical reactivity as it contributes to the formation of highly reactive radical species and underpins the chemical and antimicrobial effects associated with ultrasonic treatment. Acoustic cavitation also produces physical forces, such as microjets and shear stress in the liquid surrounding the collapsing bubble [13], while driving sonochemical reactions that generate ROS, including hydroxyl radicals (·OH), hydrogen atoms (H·), hydroperoxyl radicals (HO_2_·), and hydrogen peroxide (H_2_O_2_). Consequently, the generation of these reactive compounds is central to the bactericidal activity of ultrasound, linking cavitation dynamics directly to antimicrobial efficacy [14].

The relative contributions of physical and chemical effects during ultrasound treatment are strongly frequency-dependent [15]. LFUS (20–100 kHz) typically induces intense cavitation and pronounced mechanical effects, which can directly disrupt microbial envelopes and lead to rapid inactivation [16]. By contrast, HFUS (>100 kHz) generates smaller cavitation bubbles with less violent collapse dynamics but greater radical production, suggesting that oxidative stress and subtle membrane destabilisation become the dominant antimicrobial mechanisms at higher frequencies [17]. Despite this mechanistic distinction, most research has focused on LFUS, reflecting its established efficacy against resilient pathogens, activity against biofilms, and proven synergy with antimicrobials in both medical [18], [19] and industrial applications [20]. Nevertheless, HFUS has received comparatively limited attention [21], with most existing studies focusing on chemical transformations or advanced oxidation processes [22], and few directly addressing microbial inactivation [21], [23]. Differences in ultrasonic system configuration, including transducer design, coupling medium, and calorimetric output, further limit reproducibility and complicate cross-study comparisons [7]. Thus, these limitations highlight the need for systematic investigations conducted under controlled, indirect, and low-power conditions.

Beyond direct antimicrobial effects, ultrasound has the potential to serve as a hurdle technology when combined with other non-thermal technologies and/or bioactive substances [24]. Epigallocatechin gallate (EGCG), the predominant catechin in green tea, demonstrates various antimicrobial mechanisms, including membrane disruption and H_2_O_2_ production [25], [26]. Previous studies have demonstrated that ultrasound could enhance the penetration and antimicrobial activity of such compounds, thereby strengthening their overall antibacterial activity [27]. This enhancement occurs through mechanisms such as sonoporation, which increases membrane permeability [28], and synergistic interactions with radical mediated processes [29]. Nonetheless, the ability of HFUS to enhance the activity of antimicrobial compounds against Gram-negative bacteria remains poorly understood. EGCG was selected in the present study as a model antimicrobial molecule because its activity is often limited by the permeability barrier of the Gram-negative outer membrane [30]. Consequently, the HFUS-EGCG system provides a useful model for investigating whether HFUS-induced membrane perturbation and sonochemical effects can enhance antimicrobial efficacy.

Beyond their bactericidal potential, ultrasound may induce bacterial stress adaptation [31], which is particularly relevant for HFUS, where oxidative stress predominates [22]. Under such conditions, bacteria can activate general stress responses, sometimes acquiring cross-protection against other lethal stresses, which may contribute to antimicrobial resistance (AMR) [32]. The general stress response regulator RpoS, an alternative sigma factor that enables bacteria to survive adverse conditions, plays a central role in stress adaptation by coordinating protective pathways against oxidative and envelope stress [33]. Concurrently, redox defence systems, such as the glutathione (GSH) and thioredoxin (Trx) networks, play a primary role as first line protection against superoxide (O_2_·^–^), H_2_O_2_, and organic peroxides across many cell types by detoxifying ROS and maintaining redox balance. These adaptive response mechanisms have been shown to enhance bacterial survival following abiotic challenges such as oxidative stress, acid stress, and antibiotic treatments [34], [35], [36]. However, whether HFUS-induced ROS are sufficient to overwhelm cellular defence systems or instead trigger adaptive stress responses remains unclear due to discrepancies between existing studies [23], [37]. Clarifying whether the balance between ROS-mediated damage predominates over adaptive stress activation is therefore essential for designing effective ultrasound-based interventions without inadvertently selecting for more resilient phenotypes.

Building on previous work on ultrasonic antimicrobial mechanisms, the present study systematically investigates HFUS-induced bacterial inactivation from two complementary perspectives: the mechanistic basis of HFUS activity and the capacity of bacteria to mount adaptive responses under sublethal oxidative stress. Unlike the majority of previous studies that have focused on low-frequency, high-power ultrasound, this work examines antibacterial responses under low-power and high-frequency ultrasound conditions. For the first time, the relative contributions of chemical and physical effects were directly examined through combined radical scavenger assays and membrane disruption analyses, while EGCG was used as a model antimicrobial compound to investigate ultrasound-assisted enhancement of antibacterial activity. Alongside these mechanistic objectives, molecular-level stress adaptation responses were explored using *Escherichia coli* K-12 as a model organism, to assess whether HFUS-induced damage overwhelms cellular defences or instead activates protective pathways. By integrating mechanistic assays with mutant strain analyses, this study provides novel insights into both the oxidative and physical contributions to HFUS antimicrobial activity and the bacterial tolerance mechanisms that may limit its efficacy. These findings offer a framework to guide the development of robust, non-thermal disinfection strategies that minimise the risk of selecting for resilient phenotypes.

## 2 Materials and Methods

### 2.1 Bacterial strain and culture preparation

*E. coli* MG1655 and *E. coli* BW25113 along with single-gene deletion mutants (Table 1) from the KEIO collection (Keio University, Japan) were maintained as stock cultures at -80 °C in Tryptone Soy Broth with 0.6% of Yeast Extract (TSBYE; Oxoid Ltd., UK) supplemented with 30% glycerol. Thawed cultures were inoculated using a sterile inoculation loop into 20  mL of TSBYE and incubated in a shaking incubator for 9.5 h at 37 °C and 175 rpm. Subsequently, 20  μL of the culture was transferred into a fresh 20  mL of TSBYE and subcultured for an additional 15 h at 37 °C to reach the early stationary phase, with a final bacterial concentration of approximately 10^9^ CFU/mL as previously described [32].

**Table 1:** *E. coli* K-12 BW25113 and derived mutant strains used in this study [38].

Mutant*	Mutant Characteristics
*E. coli* K12 BW25113	Parental strain – Wild type
Δ*rpoS*	RNA polymerase σ^S (stationary phase sigma factor), global stress regulator controlling multiple stress responses
Δ*katG*	Catalase-peroxidase HPI, involved in oxidative stress response
Δ*ahpF*	Flavoprotein subunit of alkyl hydroperoxide reductase, regenerates AhpC, detoxifies peroxides
Δ*gor*	Glutathione reductase, maintains reduced glutathione pool, critical for redox balance
Δ*grxA*	Glutaredoxin 1, glutathione-dependent oxidoreductase, maintains thiol–disulfide balance
Δ*trxC*	Thioredoxin 2, maintains disulfide bond reduction under oxidative stress
Δ*ahpC*	Peroxiredoxin subunit of alkyl hydroperoxide reductase, scavenges peroxides
Δ*oxyR*	LysR-type transcriptional regulator, activates antioxidant defence genes in response to hydrogen peroxide

* All mutants were provided by the Department of Food and Nutritional Sciences, University of Reading (Kimon-Andreas Karatzas).

### 2.2 Ultrasonic conditions and experimental set-up

**Fig. 1:**
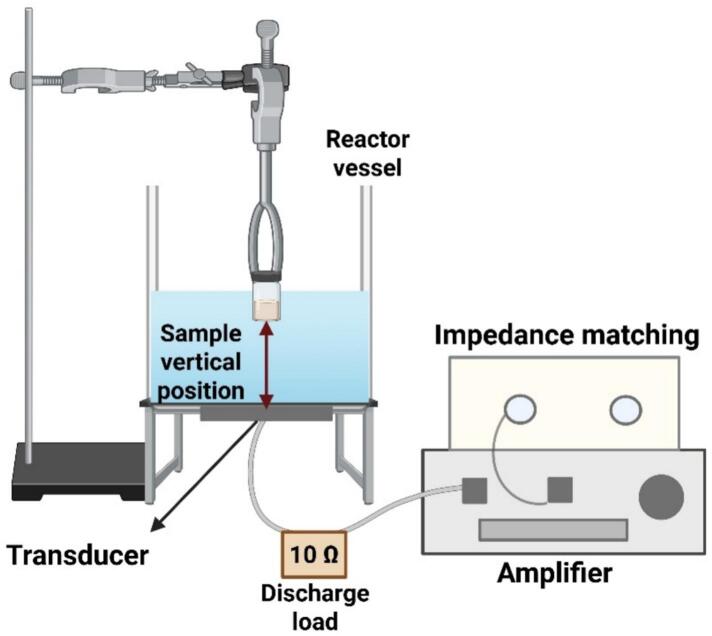
Schematic diagram of the custom-designed ultrasonic experimental set-up, comprising a piezoelectric transducer (Honda Electronics; 5 cm-diameter ceramic adhered to a 10 cm-diameter stainless steel vibration plate) mounted on a custom plastic chamber (12 × 12 × 16 cm), connected to an amplifier (T&C Power Conversion AG 1006) via an impedance matching device (IMD) and a 10 Ω discharge load to minimise reflected power. The reactor vessel contained 500 mL of carrier liquid to maximise cavitation activity while allowing sufficient immersion of the sample vial (Created by BioRender). These conditions were maintained constant across all experiments.

The custom-designed ultrasonic experimental set-up is illustrated in Fig. 1, based on a configuration reported in our previous work [7]. Interchangeable piezoelectric transducers (Honda Electronics), consisting of a 5 cm-diameter round piezoelectric ceramic adhered to a 10 cm-diameter stainless steel vibration plate, were mounted onto a custom-made plastic chamber (12 × 12 × 16 cm). The contact area between the transducer plate and the carrier liquid was 7 cm in diameter. The transducer was connected to an amplifier (T&C Power Conversion AG 1006) via an impedance matching device (IMD) and a discharge load to minimise reflected power (RP) arising from poor impedance matching. The carrier liquid volume was maintained at 500 mL to maximise cavitation activity while allowing sufficient immersion of the sample vial as informed by characterisation of ROS and temperature (Figs. S1, S2 and S3). The carrier liquid was prepared by vacuum degassing deionised water for 30 min to reduce the risk of bubble entrapment beneath the sample vial during sonication experiments. High-frequency ultrasonic transducers operating at 500 and 760 kHz were used, with an applied load power of 30 W. Frequency selection was guided by literature and preliminary experiments. Initial screening at 200, 500, and 760 kHz showed substantially lower sonochemical activity at 200 kHz, whereas 500 and 760 kHz generated comparable ROS levels as determined by KI dosimetry (Fig S1). These frequencies were therefore selected to investigate whether similar sonochemical outputs would result in different antibacterial responses and stress adaptations in *E. coli*. Sample vial vertical positioning within the reactor, which influences sonochemical activity, was also evaluated using KI dosimetry as described in Section 2.3.2.

### 2.3 Chemical characterisation of ultrasonic conditions

#### 2.3.1 Characterisation

The ultrasonic set-up was characterised via calorimetry and dosimetry. The impact of frequency and sample positioning on the sonochemical activity were examined through potassium iodide (KI) dosimetry and sonochemiluminescence (SCL) / sonoluminescence (SL) image analyses. The calorimetric power represents the ultrasonic power transferred to a solution, calculated according to Eq. 1 (Koda et al., 2003).

*P = mC_p_ (*$\frac{\mathrm{dT}}{\mathrm{dt}}$*)* (1)

Where;

*m = Mass of water (g)*

*C_p_= Specific heat capacity of water (4.19 J g^−1^ K ^-1^)*

*dT/dt = Rate of solution temperature increase during ultrasonic sonication (K s^−1^)*

Table 2 presents the calorimetric powers recorded for 500 and 760 kHz frequencies at an applied input of 30 W. Reflected power (RP) values indicate the level of energy returned from the transducer to the amplifier, which was minimised through impedance matching. Each frequency was adjusted to reduce RP and optimise energy transmission. Temperature was monitored throughout the 30 min sonication period at 30 s intervals. The corresponding temperature profiles for each frequency are provided in Fig. S1.

**Table 2:** Measurement of calorimetric powers for frequencies 500 and 760 kHz at 30 W. The reflected power (RP) values present the level of reflected power from the transducer to the amplifier. Each frequency was adjusted to avoid a high RP value resulting from poor impedance matching.

Frequency (kHz)	Applied frequency (kHz)	Reflected power (W)	Calorimetric power (W)	Calorimetric power (W/mL)	Maximum temperature (^o^C)
500	501.9	0	14.99 ± 0.21	3.00x10^-2^ ± 0.69	37.7 ± 0.49
760	762.6	2	17.35 ± 0.44	3.48x10^-2^ ± 0.36	40.4 ± 0.57

#### 2.3.2 KI dosimetry for sonochemical activity

KI Dosimetry is a standard method for evaluating sonochemical efficiency in aqueous solutions. Unlike more selective probes such as terephthalic acid, which primarily detect hydroxyl radical acids [39], KI dosimetry provides an integrated measure of the overall oxidising species generated during acoustic cavitation. Therefore, KI dosimetry was selected in the present study to compare global sonochemical activity across frequencies and sample positions rather than quantify individual radical species. The principle is based on the oxidation of iodide ions (I^–^) to triiodide ions (I_3_^–^) under ultrasonic irradiation. I_2_ molecules reversibly react with excess I^-^ to form I_3_^-^ (Eq. 5), causing the solution to turn yellow [40].

The relevant reactions (Eq. 2–5) are:

HO· + I^-^ → OH^–^ + I·(2)

I· + I- → I_2_^-^ (3)

2I_2_^-^ → I_2_ + 2I^-^ (4)

I^-^+ I_2_ ⇌ I^3-^ (5)

A 0.1 M stock KI solution was prepared by dissolving 16.6 g of powder (Fisher Scientific, UK) in 1 L deionised water and protecting from light until use. For sonochemical analyses, 10 mL of 0.1 M KI solution contained in a sample vial and positioned inside the reactor was irradiated at 30 W for up to 30 min at 500 and 760 kHz. For sample positioning experiments, the sample vial was adjusted to heights of 1.0 cm and 2.0 cm to compare the triiodide production with the reference position at 1.7 cm [7]. The selected positions encompassed the principal cavitation-active region within the reactor, as determined by KI dosimetry and SL/SCL imaging, rather than relying solely on theoretical standing-wave considerations. After treatment, the solution colour changed to yellow due to triiodide formation. The absorbance of triiodide at 355 nm was measured using a UV–Visible spectrophotometer (Thermo Scientific Evolution 201). The concentration of triiodide was calculated using the Beer–Lambert law (path length = 1 cm; molar absorptivity = 26,303 L mol^–1^ cm^–1^).

#### 2.3.3 Quantification of hydrogen peroxide (H_2_O_2_) production

KI dosimetry measures the total ROS generated, but it does not directly detect hydrogen peroxide (H_2_O_2_), as H_2_O_2_ does not react with iodide ions (I^-^) under standard conditions [41]. Therefore, a modified dosimetry method was used to quantify H_2_O_2_ by incorporating 0.5 mM ammonium molybdate into the 0.1 M KI solution, which catalyses the reaction between H_2_O_2_ and iodide [42], [43]:

*H*_2_O_2_ + 2*I* ^-^ → 2 *OH*^-^ + *I*_2_ (6)

The resulting I_2_ contributes to further triiodide formation through Eq. 6, allowing indirect quantification of H_2_O_2_. By subtracting the hydroxyl radical (HO·) contribution measured from the pure KI solution, the isolated H_2_O_2_ yield could be accurately determined.

#### 2.3.4 Sonoluminescence (SL) / sonochemiluminescence (SCL) image analyses

Sonoluminescence (SL) and sonochemiluminescence (SCL) analyses were conducted to determine the distribution of active cavitation bubbles to quantify sonochemical intensity in water and luminol solution, respectively. SL refers to the emission of light resulting from the collapse of cavitation bubbles, while SCL arises from the chemiluminescent reaction of luminol with hydroxyl radicals (HO·) generated during bubble collapse [44]. For SCL measurements, a 1 mM luminol (5-amino-2,3-dihydro-1,4-phthalazinedione, Sigma Aldrich, UK) solution was prepared with 0.1  M sodium hydroxide (NaOH, Fisher Scientific, UK) immediately prior to use. The sonication set-up was relocated to a dark room for luminescence detection. Active bubble distribution in the reactor was analysed by sonicating 350, 500, or 750 mL of either water or luminol solution at 30 W for 45 s at each frequency, with the duration standardised across all conditions to ensure consistency. For sample positioning experiments, 10 mL of luminol solution was immersed in 500 mL of degassed deionised water. Luminescence was captured using an ANDOR iXon3 EMCCD camera operated at -70 °C. Camera settings were adjusted to an EM gain of 100 and an exposure time of 20 s for SL, and an EM gain of 4 with an exposure time of 4 s for SCL. Raw images were exported as 8-bit TIFF files, and intensity values were calculated using Microsoft Excel [41]. A quantitative colour bar was not included because the images are used to visualize the spatial distribution of cavitation and identify the main light-emitting region for sample positioning, rather than to provide absolute intensity values [45]. Fresh solutions were prepared for each measurement, and a minimum of three independent repetitions were performed for both SL and SCL analyses.

### 2.4 Ultrasonic treatment and bacterial enumeration

*E. coli* MG1655 cells were cultured and grown to the stationary phase in 20 mL TSBYE broth at 37°C, as described previously (Section 2.1). The culture was serially diluted with TSBYE to decrease the initial concentration to approximately 10^8^ CFU/mL. Subsequently, 300 μL of the diluted sample was inoculated into a borosilicate scintillation vial containing 10 mL TSBYE broth, achieving a final concentration of 3x10^6^ CFU/mL. Samples were immersed in the ultrasonic reactor, (Fig. 1), and subjected to ultrasonic treatment at frequencies of 500 and 760 kHz at 30 W for 0–30 min, while varying the sample vertical positions at 1.0, 1.7, 2.0 cm. To distinguish ultrasound-specific effects from thermal effects, additional temperature control experiments were conducted by exposing bacterial samples to the maximum temperatures recorded during sonication (up to 42°C at 760 kHz) for equivalent durations in the absence of ultrasound. Following the ultrasonic treatment, samples were immediately diluted serially using 900 μL of phosphate-buffered saline (PBS). A 50 μL volume of each diluted sample was then spread-plated onto agar plates containing Tryptone Soy Agar with 0.6% of Yeast Extract (TSAYE). In parallel, untreated control samples were plated on TSAYE agar plates. After incubation at 37 °C for 24 h, colony-forming units (CFUs) were enumerated. Optical density (OD_600_) was measured in parallel to CFU enumeration to provide complementary data on bacterial growth kinetics, capturing real-time changes in cell density over the 24-h incubation period using a ClarioStar plate reader (BMG Labtech, UK). All experiments were performed in a minimum of three independent biological replicates (triplicate), each conducted with two technical replicates.

### 2.5 Assessing sonochemical contributions to bacterial inactivation

#### 2.5.1 Radical scavenger assays

To assess the contribution of sonochemically generated reactive oxygen species (ROS) to ultrasound-induced bacterial inactivation, radical scavenger assays were performed using L-histidine (Sigma-Aldrich, St. Louis, USA), a general free radical scavenger [46], and catalase (Sigma-Aldrich, St. Louis, USA), which selectively decomposes H_2_O_2_ into water and oxygen [47]. The scavenging efficiency of L-histidine was evaluated by KI dosimetry at 760 kHz, selected due to its highest ROS production at 30 W over 30 min. 10 mM L-histidine effectively suppressed radical formation, as indicated by the absence of yellow colouration and inhibition of triiodide (I_3_^–^) formation measured by UV–Vis spectroscopy (Figs. S4-S5). Catalase concentration (0.4 mg/mL; 2,000–5,000 U/mg protein) was determined based on the H_2_O_2_ levels generated during ultrasonic treatment (Section 2.3.3) and applied for 15 min. To minimise the potential thermal inactivation (∼42 °C), catalase was added immediately after ultrasound exposure, when H_2_O_2_ levels were maximal. Bacterial inactivation was assessed under four conditions: ultrasound alone, scavenger alone (L-histidine or catalase), and ultrasound combined with each scavenger, allowing differentiation between the roles of total ROS and H_2_O_2_ in ultrasound-mediated bacterial inactivation.

#### 2.5.2 Evaluation of intracellular ROS generation

To quantify intracellular ROS levels in *E. coli*, 100  μL ultrasonic-treated and control bacterial suspensions were incubated with 10  μM 2′,7′-dichlorodihydrofluorescein diacetate (DCFH-DA; Sigma-Aldrich, UK) for 30  min at 37 °C in the dark, as described previously [48]. Fluorescence was measured using an Attune™ NxT Flow Cytometer equipped with a 488  nm solid-state laser, with detection in the BL1 channel (530/30 nm filter). Three independent biological replicates were performed. Flow cytometry (FC) data were processed using FlowJo™ software (version 10.10.0; BD Life Sciences). For plate reader measurements, samples were washed after incubation to remove excess DCFH-DA and resuspended in PBS. Fluorescence intensity (Ex/Em: 485/530  nm) was recorded using a CLARIOstar plate reader (BMG LABTECH), and values were corrected against corresponding blank controls to ensure accurate quantification of intracellular ROS.

### 2.6 Assessment of ultrasound-induced physical effects

#### 2.6.1 Epigallocatechin gallate (EGCG)-assisted bacterial inactivation under ultrasonic treatment

Epigallocatechin gallate (EGCG; Sigma-Aldrich, UK) is a bioactive compound derived from green tea extract, recognised for its antimicrobial properties [30]. EGCG was applied in combination with ultrasound at 760 kHz and 30 W to investigate whether the combination would enhance antimicrobial efficacy at this frequency. To determine a sublethal concentration of EGCG against *E. coli*, bacterial suspensions were treated with varying concentrations of EGCG (0.0125, 0.2, 0.4, 0.6, and 1 mg/mL). Samples were incubated at 37°C, and OD_600_ was measured every 10 min using a ClarioStar plate reader (BMG Labtech, UK). A concentration of 0.2  mg/mL EGCG was identified as sublethal for *E. coli* and was therefore selected to assess its combined effect with ultrasound treatment (Fig. 7a). The use of a sublethal concentration ensured that any enhanced antimicrobial activity observed could be attributed to the interaction between EGCG and ultrasound, rather than to individual treatments. EGCG stability under ultrasonic exposure (760  kHz, 30  W, 30  min) was evaluated, revealing only ∼ 5% degradation at the μg-per-mL level, indicating minimal compound loss during the treatment (Fig. S6). Combination treatments were applied in two different sequences: (i) EGCG treatment (0.2 mg/mL) for 30 min followed by ultrasound exposure at 760 kHz and 30 W for 30 min, and (ii) ultrasound treatment followed by EGCG application.

#### 2.6.2 Bacterial cell membrane integrity assay

Membrane integrity was assessed following a previously described method [49] with minor modifications. Overnight cultures (1 mL) were centrifuged at 8000 × g for 5 min, washed twice with PBS, and resuspended in 1 mL PBS and 9 mL sterile Milli-Q water to obtain a final concentration of 10^8^ CFU/mL prior to ultrasonic and combination treatments. After treatment, 100 μL of bacterial suspension was mixed with 900 μL PBS containing Bis-(1,3-dibarbituric acid)-trimethine oxonol (DiBAC_4_(3); 50 ng/mL) and propidium iodide (PI; 4 μg/mL). Samples were incubated in the dark for 15 min at room temperature. DiBAC_4_(3), a voltage-sensitive dye, and PI, a membrane-impermeant nucleic acid stain, were used to indicate membrane depolarisation and permeabilisation, respectively. Samples were analysed immediately using an Attune™ NxT flow cytometer (488 nm laser), with DiBAC_4_(3) detected via the 530/30 BP (BL1) channel and PI via the 620/15 BP (YL2) channel. Data were analysed using FlowJo™ software (v10.10.0), and corresponding untreated samples were used as controls.

Membrane disruption was further evaluated by quantifying DNA/RNA and protein leakage into the supernatant by measuring absorbance at 260 nm and 280 nm, respectively, using a UV–Vis spectrophotometer (Thermo Scientific, UK), as previously described [50]. Following treatments, samples were centrifuged at 4600 × g for 10 min at 4 °C, and supernatants were analysed. A second centrifugation step was performed when necessary to minimise contamination from residual cells.

#### 2.6.3 Scanning electron microscopy (SEM) imaging of bacterial cell damage

1 mL of overnight culture was transferred into 9 mL TSBYE and subjected to ultrasonic treatment at 500 and 760 kHz for 30 min at 30 W and EGCG (0.2 mg/mL). Samples not exposed to ultrasound underwent the same procedures and were used as controls (silent treatment). After treatment, all samples were centrifuged at 4500 × g for 10 min at 4°C to collect cell pellets. The pellets were washed twice with Dulbecco’s Phosphate-Buffered saline (DPBS; Corning, UK) modified to exclude CaCl_2_ and MgCl_2_, and subsequently resuspended in 1 mL of DPBS and 1 mL of 3% (v/v) formaldehyde solution to fix the cells for 1 h at room temperature. Following fixation, the volume was adjusted to 5 mL with DPBS, and the suspension was passed through a sterile 13 mm diameter polycarbonate track-etched membrane filter (0.22 μm pore size, GE Healthcare Whatman, Fisher Scientific, UK) held in a sterile Swinnex filter holder (13 mm, Merck, USA). Samples underwent serial dehydration steps using ethanol concentrations of 20%, 40%, 60%, 80%, and 100%, with each step involving a 10-min incubation; the final 100% ethanol step was repeated twice at room temperature. The filters were allowed to air-dry overnight before being mounted onto aluminium stubs using carbon conductive tape. Samples were then sputter-coated with a 3 nm gold layer using an EMITECH K575X sputter coater (Quorum Technologies, Lewes) prior to imaging. Samples were visualised using an Apreo scanning electron Microscope (Thermo Fisher Scientific, UK) operated at an accelerating voltage of 5 kV and a beam current of 0.2nA. All images were acquired at a scale of 2 μm. SEM analysis was performed on samples obtained from three independent biological experiments, and at least six representative images were acquired for each treatment. Raw images were analysed by using ImageJ (1.54g, USA)

### 2.7 Stress adaptation after ultrasound

#### 2.7.1 Survival in the presence of lethal dose of H_2_O_2_ and heat

**Fig. 2:**
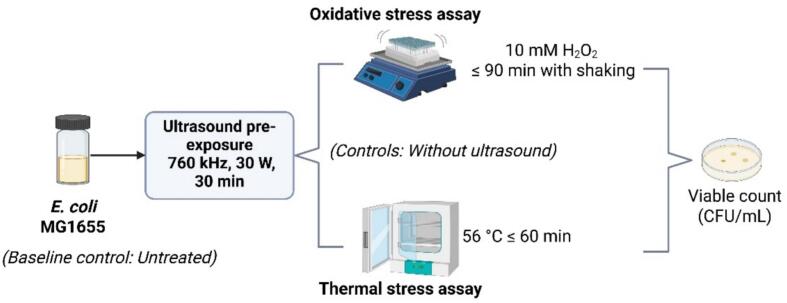
Schematic overview of the experimental design used to assess the tolerance of ultrasound pre-exposed *E. coli* MG1655 to oxidative and thermal stress. Following ultrasound treatment at 760 kHz and 30 W for 30 min, cells were challenged with H_2_O_2_ at 10 mM or heat at 56 °C. Control groups included untreated cells and stress treatments applied in the absence of ultrasound.

To assess the tolerance of ultrasound-exposed *E. coli* MG1655 to oxidative and thermal stress under lethal conditions, overnight cultures were diluted in TSBYE to a final concentration of 3 × 10⁶ CFU/mL. Samples of 10 mL were exposed to ultrasound at 760 kHz and 30 W for 30 min. Following sonication, cells were challenged either with 10 mM H_2_O_2_ at room temperature under shaking conditions for maximum 90 min or with heat treatment at 56 °C for 60 min (Fig. 2). These challenge conditions were chosen to impose strong but non immediate oxidative or thermal stress, enabling progressive inactivation while allowing evaluation of stress tolerance and cross protection effects following ultrasound exposure [51], [52]. Cells were either pre-exposed to ultrasound or left unsonicated, then subjected to lethal oxidative stress (10 mM H_2_O_2_) or thermal stress (56 °C). Untreated cells served as the baseline control, while H_2_O_2_-only and heat-only treatments without ultrasound were used as corresponding stress controls. After treatment, samples were serially diluted, plated on TSAYE, and incubated at 37 °C for 24 h before colony enumeration. All experiments were performed with at least three independent biological replicates.

#### 2.7.2 Analysis of genetic deletion strains under ultrasonic treatment

To investigate the molecular bacterial responses to ultrasonic-induced stress, eight *E. coli* K-12 single-gene deletion mutants from the KEIO collection were used (Table 1), along with the wild-type strain *E. coli* K-12 BW25113. The selected mutants were in genes involved in key oxidative stress response pathways (*oxyR*, *katG*, *ahpC*, *ahpF*), redox regulation (*gor*, *grxA*, *trxC*), and the general stress response regulator *rpoS*. Frozen stocks in 50 μg/mL kanamycin were thawed, and overnight cultures were prepared as described in Section 2.1. Subsequently, 300 μL of the diluted sample of 10^8^ CFU/mL was inoculated into a borosilicate scintillation vial containing 10 mL TSBYE broth, achieving a final concentration of 3x10^6^ CFU/mL. Each strain was either left untreated or exposed to HFUS (760 kHz, 30 W) for 30 min using the ultrasonic set-up described previously (Section 2.4). After treatment, bacterial suspensions were serially diluted and plated onto TSAYE for colony enumeration. All experiments were performed in at least three biological triplicates.

### 2.8 Statistical analysis

Unless otherwise stated, all experiments were conducted using at least three independent biological replicates, and each biological replicate was analysed in duplicate to ensure technical reliability. Statistical analyses were performed using analysis of variance (ANOVA) in Jamovi version 2.44.6.0 (Australia) to assess significant differences among groups based on ultrasonic frequency and sample vertical position for triiodide production and bacterial inactivation. When ANOVA indicated significance, Tukey’s post-hoc test was applied for multiple comparisons between group means at a significance threshold of *p* ≤ 0.05. Additionally, unpaired-sample t-tests were conducted using GraphPad Prism version 10.1.0 (USA) to evaluate specific treatment effects on bacterial inactivation. Differences were considered statistically significant at *p < 0.05, **p < 0.01, and ***p < 0.001. All results are presented as mean values ± standard deviation (SD).

## 3 Results and discussion

The antimicrobial effects of HFUS and the associated bacterial responses under controlled, low power conditions are presented and discussed in this section. The results are organised around three central objectives; i) to define the physicochemical drivers of ultrasound activity (Section 3.1), ii) to evaluate bacterial inactivation under ultrasound alone (Section 3.2) and in combination treatments (Section 3.4), and iii) to determine the relative contributions of oxidative (Section 3.3) and physical mechanisms (Section 3.4), including the potential for adaptive responses and cross-protection (Sections 3.5 - 3.6).

### 3.1 Parametric effect of ultrasound on sonochemical activity

**Fig. 3:**
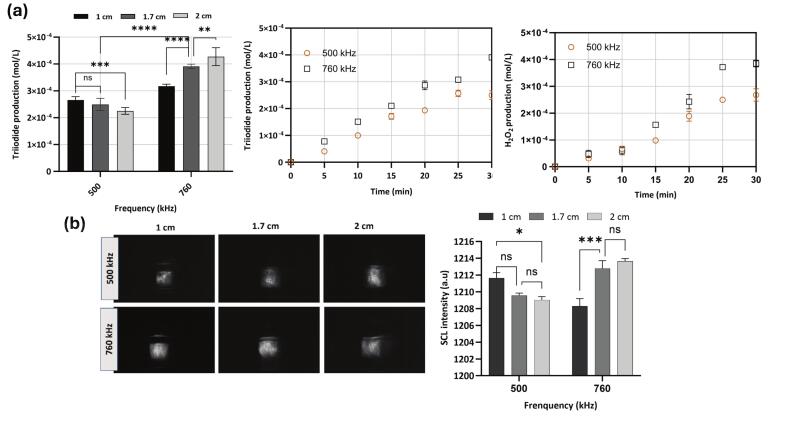
Sonochemical characterisation of the ultrasonic system at 30 W. (a) KI dosimetry and H_2_O_2_ measurements: Triiodide production at different sample positions (1, 1.7, 2 cm) after 30 min of sonication (left); Time-dependent triiodide production at 500 and 760 kHz (middle); H_2_O_2_ generation over time at 500 and 760 kHz (right). Statistically significant differences are denoted as **p < 0.01, ***p < 0.001 and ****p < 0.0001 (n ≥ 3 independent repeats). (b) Sonochemiluminescence (SCL): Representative SCL images in 10 mL luminol solution (in 500 mL water) at different heights and frequencies (left); Quantified SCL intensity across sample positions (right). Data are presented as mean ± standard deviation, with error bars indicating standard deviation (SD).

Before assessing the antimicrobial efficacy of the custom-designed ultrasonic system, sonochemical performance was characterised by assessing energy delivery and cavitation behaviour under different operational conditions. Calorimetric measurements at a constant applied power of 30 W revealed that the highest acoustic power was achieved at 760 kHz (17.35 ± 0.44 W) (Table 2), indicating a more efficient energy transfer at higher frequencies. To optimise the system set-up, triiodide ion generation via KI dosimetry was initially used to identify the most active cavitation zones across different vertical sample positions. Distinct optimal positions emerged for each frequency, with triiodide production increasing with height at 760 kHz but decreasing at 500 kHz (Fig. 3a). This opposite trend is likely attributable to frequency-dependent differences in the spatial distribution of cavitation activity within the reactor. Supporting this interpretation, SL and SCL imaging of the entire reactor (Figs. S2 and S3) revealed that the most active cavitation region at 760 kHz was located closer to the liquid surface, whereas cavitation activity at 500 kHz was more broadly distributed throughout the reactor. These findings emphasise the spatial dependency of cavitation behaviour and the need to define the acoustic active region in this set-up. This frequency-position interaction was further supported by SL and SCL imaging, which revealed analogous spatial patterns in cavitation intensity (Fig. 3b). At 500 kHz, KI/SCL activity was maximal at 1 cm and remained comparable at 1.7 cm, before significantly decreasing at 2 cm. Conversely, at 760 kHz, KI/SCL activity increased with distance from the transducer, with the highest values observed at 2 cm, although these were not significantly different from those at 1.7 cm. These results confirm that the location of maximum sonochemical activity is frequency dependent in this system, highlighting the critical role of spatial characterisation for ultrasonic applications (Fig. 3b).

Combining physical (SL), chemical (SCL), and spatial (KI dosimetry) analyses provided a comprehensive view of cavitation dynamics within the reactor. Based on these multi-modal observations, a vertical sample position of 1.7 cm above the transducer was chosen as the optimal height for subsequent experiments, enabling consistent comparison across frequencies. To statistically validate these spatial and frequency-dependent effects, a two-way ANOVA was conducted on KI dosimetry results. The analysis revealed a significant main effect of frequency (*p* < 0.0001, η^2^ = 74.38%) and sample position (*p* < 0.0001, η^2^ = 3.92%), along with a significant interaction between the two variables (*p* < 0.0001, η^2^ = 16.45%), confirming that sonochemical activity was strongly influenced by both parameters and their interaction (η^2^: proportion of variance, Tables S1 and S2). These findings support the empirical observation that optimal cavitation zones vary by frequency and justify the use of 1.7  cm as a common effective position across different frequencies.

Using the selected sample positioning (1.7 cm above the transducer), sonochemical activity was further evaluated over time by monitoring triiodide ion and H_2_O_2_ generation during 5 to 30 min of sonication at each frequency. Triiodide production increased with time, with significantly higher yields at 500 and 760 kHz, corresponding to ROS production rates of 8.0 × 10^–^⁶ and 1.07 × 10^–5^ mol·L^–1^·min^–1^, respectively (Fig. 3a). Likewise, H_2_O_2_ concentrations were consistently higher at 760 kHz, reaching approximately 4.0 × 10^–4^ mol·L^–1^ after 30 min (Fig. 3a). These findings establish a physicochemical baseline by demonstrating that HFUS, under characterised experimental conditions and defined sample positioning, enhances cavitation intensity and sonochemical activity. This baseline provides the necessary framework to examine antibacterial efficacy in subsequent analyses and to interpret antimicrobial outcomes in relation to sonochemical activity.

### 3.2 Impact of characterised ultrasonic conditions on antibacterial efficacy

**Fig. 4:**
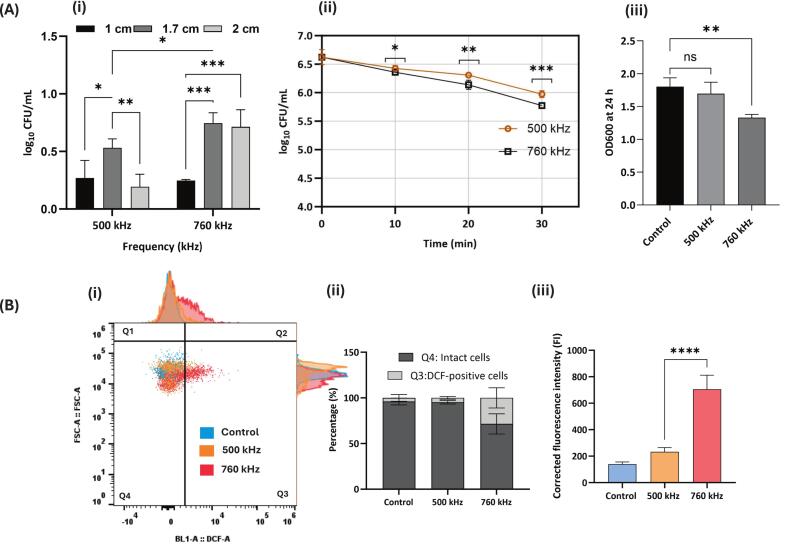
HFUS-induced antibacterial activity and oxidative stress responses in *E. coli*. (A) Antibacterial efficacy following ultrasonic treatment: (i) effect of sample position (1.0, 1.7, and 2.0 cm) at 500 and 760 kHz; (ii) bacterial inactivation kinetics over 30 min at 1.7 cm; and (iii) bacterial regrowth after 24 h measured by OD_600_. (B) Intracellular ROS production following ultrasonic treatment at 500 and 760 kHz (30 W, 30 min, 1.7 cm): (i) representative flow cytometry fluorescence profiles; (ii) quantification of intracellular ROS based on flow cytometry analysis; and (iii) ROS quantification using a fluorescence plate reader. Bars represent mean ± SD of three independent biological replicates. Statistically significant differences are denoted as *p < 0.05, **p < 0.01, ***p < 0.001, ****p < 0.0001, and ns, not significant (n ≥ 3 independent repeats).

To evaluate ultrasound-mediated bacterial inactivation, planktonic *E. coli* MG1655 was treated under the previously identified conditions (Section 3.1). Sample positioning had a significant impact, with the highest inactivation observed when the sample was placed 1.7 cm above the transducer at both 500 and 760 kHz. However, 760 kHz consistently outperformed 500 kHz, particularly at 1.7 and 2.0 cm (Fig. 4A(i)). Despite the distinct KI/SCL activity profiles observed across reactor heights, the maximum bacterial inactivation at both frequencies consistently occurred at 1.7 cm, indicating that bacterial reduction was not solely correlated with sonochemical activity measurements. These findings suggest that bacterial inactivation was governed not only by bulk ROS generation, but also by localised cavitation-associated effects, including mechanical disruption and localised thermal stresses [53], which may have been more pronounced at 1.7 cm.

Using 1.7  cm as the standard position, the effect of frequency over time was further examined. 760  kHz induced a higher time-dependent reduction in viability than 500 kHz, with significant differences observed (Fig. 4A(ii)). After 30 min, bacterial reduction reached ∼0.85-log at 760  kHz compared to ∼0.65-log at 500  kHz (*p* < 0.05). Consistent with this, OD_600_ measurements after 24 h of regrowth were lower for the 760 kHz treatment than for both 500 kHz and the control, indicating a more persistent inhibitory effect (Fig. 4A(iii), Fig. S7). At higher frequencies, cavitation bubble collapse becomes less violent but radical production increases, suggesting that oxidative stress and membrane destabilisation are the dominant antimicrobial mechanisms within this frequency range [15], [17]. The increased bacterial inactivation observed at 760 kHz is likely associated with the higher levels of ROS and H_2_O_2_ generated at this frequency (Fig. 3a), yet the distinct stress adaptation responses recorded in *E. coli* suggest that factors beyond total ROS quantity, including radical species distribution and localised acoustic intensity, may also differentially influence bactericidal outcomes at 500 and 760 kHz. These observations align with previous studies reporting a strong frequency dependence of HFUS-mediated bacterial inactivation. Costello et al. [7] observed approximately a 1-log reduction in planktonic *E. coli* at 500 kHz using HFUS at 30 W, whereas little to no inactivation occurred at 1000 kHz under the same conditions. Similarly, Hua & Thompson [54] reported decreasing *E. coli* inactivation with increasing frequencies from 205 to 1071 kHz at 128 W in oxygenated systems. These findings suggest that effective HFUS-mediated inactivation occurs within a limited frequency range where acoustic cavitation and radical formation remain efficient. Frequencies between approximately 300 and 500  kHz have been reported as particularly productive in sonochemical activity [55], whereas higher frequencies (e.g., 583–1140 kHz) are associated with reduced bubble collapse intensity and shorter bubble growth times, resulting in diminished cavitation effects [56], [57]. However, these effects are strongly dependent on the reactor configurations, thus baseline studies that link acoustic parameters to reactor geometry and energy distribution are required to reliably interpret and compare sonochemical performance across different systems [57], [58].

Calorimetric power reflects the effective acoustic energy transferred to the medium and has been linked to antimicrobial efficacy [59]. The lower energy input at 500 kHz (14.99 ± 0.21 W) likely accounts for the reduced inactivation observed under tested conditions. Previously, higher calorimetric power has been associated with enhanced microbial inactivation, including effective inactivation of planktonic bacteria [59] and partial inactivation of more resilient spores such as *Bacillus subtilis* [60] and *Clostridium butyricum* [61]. In the present study, operation at 760 kHz resulted in greater calorimetric power transfer to the liquid bulk (19.1 ± 1.6 W) and higher heat transfer, with temperatures reaching approximately 42 °C. These conditions coincided with enhanced bacterial inactivation. The temperature was not externally controlled during treatments to better reflect realistic processing conditions. Importantly, exposure to the maximum ultrasound-induced conditions (42 °C and 0.4 mM H_2_O_2_), applied individually or in combination without ultrasound, did not produce inactivation levels comparable to ultrasound treatment alone (Fig. S1C), confirming that antibacterial efficacy cannot be attributed to these factors independently. Nevertheless, heat is well known to act synergistically with ultrasound when applied concurrently, a combined effect commonly described as thermosonication [62]. The combined influence of increased temperature and higher ultrasonic frequency likely contributed to enhanced sonochemical activity [63] and increased bacterial reduction, consistent with previous reports [14], [60].

Previously, HFUS inactivation mechanisms have been primarily associated with sonochemical effects driven by hydroxyl radicals and H_2_O_2_ [16], whereas LFUS has been linked mainly to physical effects [9]. However, the observed correlation between enhanced sonochemical activity, reflected by higher calorimetric power and temperature rise, increased ROS production, and greater reductions in *E. coli* viability suggests a combined contribution of ROS-mediated and physical mechanisms rather than a single dominant inactivation pathway. This combined behaviour can be explained by the fact that elevated temperature and calorimetric power enhance sonochemical activity while simultaneously intensifying cavitation-related physical effects such as shear forces and microstreaming that perturb bacterial cell envelopes [9]. These observations highlight the importance of distinguishing and, where possible, quantifying the relative contributions of ROS-driven and physical effects to clarify HFUS-based antimicrobial mechanisms and maximise bactericidal efficacy.

### 3.3 Intracellular ROS production and the role of ROS in bacterial inactivation

ROS act as bactericidal agents that can reduce cell viability and cause damage to proteins and DNA [29]. Intracellular ROS generation following ultrasonic treatments at 500 and 760 kHz (30 W, 1.7 cm sample height, 30 min) was assessed using FC based on fluorescence production and further quantified using a plate reader. The DCFH-DA probe, which is oxidised to the fluorescent compound DCF by ROS, showed increased fluorescence, confirming ROS generation (Fig. 4B(i)). Following exposure to 760 kHz ultrasound, a significant increase in intracellular ROS was observed (DCF-positive cells in Quadrant 3 (Q3) − Fig. 4B(ii)). This increase was further validated by plate reader measurements, where Fig. 4B(iii) shows the relative fluorescence intensity of the 485/530 nm signal, reflecting intracellular ROS levels. While 500 kHz exposure resulted in a slight increase in ROS compared to the control, 760 kHz induced significantly higher ROS accumulation than both 500 kHz and control treatments, confirming FC results.

The notably elevated intracellular ROS levels detected at 760 kHz suggest that ROS-mediated inactivation contributes substantially to bactericidal activity at this frequency, which is consistent with previous studies linking HFUS to intensified sonochemical ROS generation [46], [64]. In contrast, although comparable levels of ROS generation were recorded at 500 kHz (Fig. 3a), the lower intracellular ROS accumulation highlights the involvement of additional inactivation mechanisms beyond oxidative stress alone. This frequency-dependent impact suggests although HFUS at 500 kHz can trigger substantial ROS generation, the intracellular ROS levels may be insufficient to cause substantial oxidative damage. By comparison, the marked increase in intracellular ROS signal at 760 kHz (Fig. 4B) likely reflects not only enhanced ROS production but also increased membrane permeabilisation, facilitating ROS translocation into the cytoplasm. Accordingly, differences in intracellular ROS levels strengthen the view that HFUS-mediated bacterial inactivation may not be solely driven by ROS or H_2_O_2_ but also involves membrane permeability changes and related physical effects [7] that may vary with resonance bubble size [65].

**Fig. 5:**
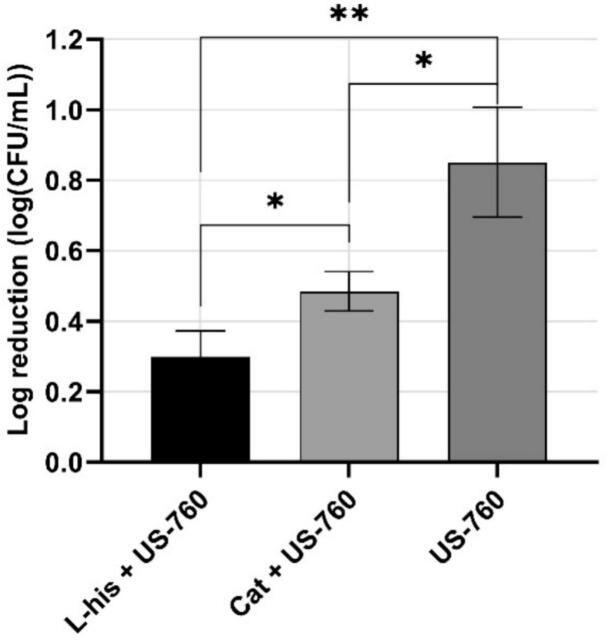
*E. coli* bacterial reduction following treatment with ultrasound (US-760  kHz, 30  W, 30  min) alone and in combination with L-histidine (L-His) (10  mM) or catalase (Cat) (0.4  mg/mL). Data represent mean ± SD of three replicates. Statistically significant differences are denoted as *p < 0.05 and **p < 0.01 (n ≥ 3 independent repeats).

To elucidate the contribution of ROS to HFUS-induced bacterial inactivation, specific scavengers were incorporated during 760 kHz treatment and *E. coli* survival measured (Fig. 5). The bacterial inactivation was significantly lower in the presence of L-histidine (10  mM) and catalase (0.4 mg/mL) compared to ultrasound treatment alone (US-760). The more pronounced reduction in bacterial inactivation observed in the presence of L-histidine compared to catalase suggests that highly reactive and short-lived species such as ^1^O_2_, ·OH, and O_2_·^–^ may contribute more substantially to bacterial inactivation than H_2_O_2_. Consistent with our findings, Zhang et al. [29] reported that bacterial inactivation of *E. coli* under ultrasonic treatment (20 kHz, 90 W/L) was significantly reduced by the addition of benzoquinone and t-butanol, scavengers of short-lived reactive species. Their study attributed ultrasound-induced inactivation primarily to radicals generated by acoustic cavitation, including O_2_·^–^/HO_2_· and ·OH, while H_2_O_2_, evaluated using catalase to enzymatically decompose it into water and oxygen, was identified as a secondary contributor.

The contribution of ROS to ultrasound-mediated bacterial inactivation has been highlighted in previous studies. For instance, Dolan et al. [46] reported a significant role of ROS at 20 kHz. However, their work combined LFUS with zinc oxide, a compound that itself promotes oxidative stress, making it difficult to attribute the observed inactivation solely to ultrasound-induced ROS. In contrast, Gao, Hemar, et al. [23] provided clearer evidence for ROS involvement at high frequency (850 kHz). Using t-butanol as a scavenger, they confirmed that ROS played a major role in the inactivation of *Enterobacter aerogenes*, *Bacillus subtilis*, and *Staphylococcus epidermidis*. Transmission electron microscopy (TEM) images further supported this conclusion, showing minimal membrane disruption and indicating that bacterial reduction was largely the result of chemical interactions between ROS and the cells rather than direct physical damage. Notably, *E. aerogenes*, one of the organisms tested, is Gram-negative as is *E. coli*, suggesting some structural basis for comparison, although direct extrapolation across species would require further investigation.

### 3.4 Mechanical damage by HFUS

#### 3.4.1 Synergistic antibacterial effect of EGCG and ultrasound with SEM analysis of membrane damage

**Fig. 6:**
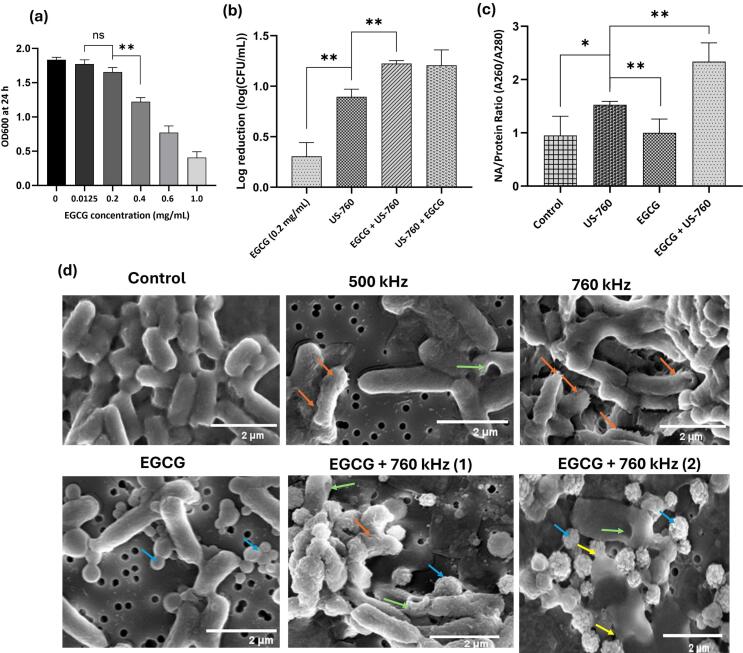
Antibacterial effect of EGCG and ultrasound treatments on *E. coli*: (a) Effect of different EGCG concentrations on bacterial growth after 24 h (b) Inactivation levels and (c) nucleic acid/protein leakage (d) SEM visualisation (2 µm) of cell membrane disruption, showing visible cell surface deformations (orange arrows), membrane pore formation (green arrows), EGCG residues on cell and filter surfaces (blue arrows), and clustered EGCG aggregates adhered to bacterial surfaces in ultrasonic-treated samples (yellow arrows). Bars represent mean ± SD of at least three replicates. Statistically significant differences are denoted as *p < 0.05, **p < 0.01, ***p < 0.001 and *ns*: not significant.

To assess the mechanical contribution of HFUS to bacterial inactivation, the potential of enhanced access and combined treatments were examined using EGCG (0.2  mg/mL) as a model antimicrobial compound. This concentration was selected as a sublethal treatment level based on preliminary concentration screening (0.0125–1 mg/mL; Fig. 6a), enabling the contribution of HFUS to be evaluated without substantial bacterial inhibition by EGCG alone. Bacterial samples were subsequently analysed with SEM to visualise membrane alterations and EGCG interactions.

While EGCG or 760 kHz ultrasound treatment alone resulted in modest reductions in viable *E. coli* counts (∼0.3 log and ∼0.85 log CFU/mL, respectively), their combination produced a ∼1.2 log reduction (Fig. 6b), indicating enhanced antibacterial activity compared with either treatment alone. This is consistent with previously reported combined treatments of HFUS with natural antimicrobial compounds, where similar or greater incremental improvements have been documented [7], [8]. For example, Zhang et al. [8] reported that combined treatment with 1 MHz ultrasound and 10 mM citral generated greater than 1.5 log CFU/mL additional inactivation of *E. coli* K12. The moderate magnitude of enhancement observed here is likely reflective of the sublethal EGCG concentration employed (0.2 mg/mL), which was deliberately selected to enable investigation of stress responses rather than maximise inactivation. To quantify membrane damage, the nucleic acid to protein (NA/protein) release ratio was measured (Fig. 6c). The combination treatment (EGCG + US-760  kHz) yielded the highest ratio, suggesting substantial leakage of intracellular contents and considerable membrane disruption (Fig. 6c). Notably, 760  kHz ultrasound alone produced greater leakage than 500  kHz, aligning with earlier observations of increased cavitational activity (Fig. 3b) and antibacterial effects (Fig. 4A).

The biochemical indicators of cell envelope damage were supported by SEM (Fig. 6d). Structural alterations were evident across all ultrasound-treated groups, with visible cell surface deformations (orange arrows) and membrane pore formation (green arrows) particularly prominent under the EGCG + US-760  kHz treatment (Fig. 6d). In addition, EGCG residues were visible on cell surfaces (blue arrows), while more clustered EGCG aggregates were found adhered to the bacterial membranes in ultrasonic-treated samples (yellow arrows), suggesting ultrasound-enhanced interaction between EGCG and the bacterial envelope. Notably, these interactions appeared specific to bacterial cells, as no EGCG residues were observed on non-bacterial surfaces (i.e. filter paper), regardless of ultrasonic treatment (Fig. S8). This suggests that bacterial surfaces provide binding sites for EGCG, facilitating its accumulation on the membranes [66], [67]. Overall, the leakage and SEM data support enhanced membrane disruption and increased EGCG-bacterial surface interactions as key contributors to the improved antibacterial efficacy of the HFUS-EGCG treatment, although additional intracellular and oxidative mechanisms cannot be excluded.

The increased attachment of ECGC to bacterial cells following ultrasound exposure may be explained by the ability of HFUS treatment to transiently disrupt bacterial barriers through enhanced membrane fluidity, depolarisation, or temporary pore formation (Fig. 6d), thereby enhancing EGCG accessibility and attachment. Similarly, the combination of ultrasound (500 kHz) and nisin (35 IU/mL) has been shown to exploit this mechanism, whereby transient pore formation induced by ultrasound facilitates nisin uptake into the cell [7]. Mechanistically, EGCG binds to peptidoglycan in Gram-positive bacteria, causing membrane disruption [68]. Its effectiveness against Gram-negative bacteria, such as *E. coli*, is typically limited due to the protective outer membrane and negatively charged lipopolysaccharides (LPS), which hinder EGCG interaction [69]. SEM images revealed that EGCG particles appeared more granulated or aggregated following ultrasound treatment, suggesting possible conformational or physical changes induced by ultrasonication. These morphological modifications may enhance surface interaction between EGCG and bacterial membranes, promoting stronger binding or localised accumulation (blue arrows). Such structural modifications have been previously reported in other ultrasound-assisted antimicrobial systems. For example, Ma et al. [70] showed that 20 kHz ultrasound altered the surface properties of whey protein-totarol nanoparticles, significantly increasing their negative zeta potential. This increase in surface charge suggests changes in molecular conformation and electrostatic interactions, which could enhance antimicrobial effectiveness. Moreover, previous studies have suggested that ultrasonication may modify EGCG structure through cavitation-induced oxidation and hydrogen bond disruption, leading to shifts in hydroxyl and aromatic group vibrations and potential formation of quinone-like structures [71], [72]. Such changes could influence EGCG’s surface activity and interaction with bacterial membranes.

These findings support the hypothesis that HFUS promotes a synergistic antibacterial impact not only by compromising bacterial membrane integrity but also by enhancing compound-membrane interactions. The observed synergistic inactivation of bacteria by EGCG combined with 760 kHz ultrasound highlights the potential of ultrasound-assisted antimicrobial strategies to overcome the intrinsic resistance of Gram-negative pathogens to polyphenolic agents. For instance, Liu et al. [73] reported that combining nano-emulsified basil essential oil with 20  kHz, 900  W ultrasound significantly enhanced its antibacterial activity against *Salmonella enterica* spp., further demonstrating the synergistic potential of ultrasound in boosting otherwise limited antimicrobial effects.

Although EGCG is widely recognised as an antioxidant and radical scavenger, increasing evidence suggests that it may also exhibit pro-oxidant antibacterial activity depending on treatment conditions and concentration [74]. Previous studies in *E. coli* demonstrated that EGCG-induced bacterial inactivation was associated with increased intracellular ROS accumulation and oxidative stress rather than ROS suppression [75]. Notably, Xiong et al. further reported that EGCG showed synergistic bactericidal activity when combined with paraquat, a superoxide-generating oxidative stress compound, while its antibacterial effect was abolished under anaerobic conditions, supporting the role of oxidative mechanisms in EGCG-mediated inactivation. Similarly, Cui et al. [76] demonstrated that combined EGCG and cefotaxime treatment significantly increased intracellular oxidative stress compared to either treatment alone, suggesting cooperative interactions between exogenous and endogenous ROS generation. In the present study, EGCG reduced triiodide formation during KI dosimetry, indicating partial scavenging of sonochemically generated oxidising species in the surrounding medium (Fig. S6). However, no significant changes in intracellular ROS accumulation were observed when EGCG was applied either before or after HFUS treatment. Despite this apparent reduction in extracellular sonochemical activity, enhanced bacterial inactivation was still observed during combined HFUS-EGCG treatment. These findings suggest that the observed synergy cannot be explained solely by ROS accumulation and that additional mechanisms, including HFUS-induced membrane perturbation and enhanced bacterial susceptibility to EGCG, may contribute to the antibacterial response.

Nevertheless, the present work was not designed to comprehensively characterise interactions between EGCG and individual HFUS-generated reactive species, nor to assess the influence of different EGCG concentrations and treatment sequences. Therefore, the precise interplay between HFUS-generated oxidative stress, membrane permeability changes, EGCG-associated effects, and intracellular stress responses remains unclear and warrants further investigation using targeted intracellular and extracellular mechanistic approaches.

#### 3.4.2 Membrane disruption patterns by flow cytometry (FC)

To further investigate if the observed cell damage involved membrane depolarisation and permeabilisation, FC analyses were conducted using specific fluorescent probes. These analyses provided additional insight into membrane integrity. Ultrasound at 760 kHz application resulted in elevated membrane permeabilisation along with moderate depolarisation, while 500 kHz mainly caused depolarisation with limited permeabilisation This restricted membrane permeabilisation at 500 kHz may underlie the reduced bacterial inactivation observed relative to 760 kHz. Although KI dosimetry indicated comparable overall sonochemical activity at both 500 and 760 kHz (Fig. 3a), intracellular ROS levels were markedly higher at 760 kHz (Fig. 4B). One possible explanation is that the greater membrane disruption observed at 760 kHz facilitated increased intracellular exposure to oxidative stress. However, membrane permeability alone may not fully explain this discrepancy. Differences in extracellular ROS dynamics, localised sonochemical microenvironments [77], and frequency-dependent cavitation behaviour [56], [57] may also influence intracellular ROS accumulation. Therefore, the higher intracellular ROS levels observed at 760 kHz likely reflect the combined effects of membrane perturbation and complex sonochemical processes rather than a single underlying mechanism.

**Fig. 7:**
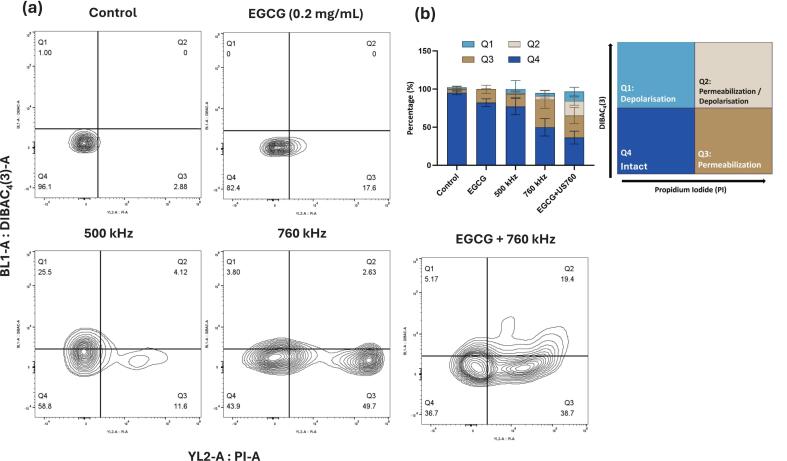
The impact of ultrasound frequency and EGCG treatment on *E. coli* membrane integrity evaluated by flow cytometry. (a) Representative flow cytometry plots showing fluorescence signal shifts after staining with DiBAC_4_(3) (y-axis, membrane depolarisation) and propidium iodide (PI; x-axis, membrane permeabilisation). Cells were exposed to ultrasound at 500 or 760 kHz, EGCG (0.2 mg/mL), or EGCG and 760 kHz combinations for 30 min at 30 W. Untreated cells were used as the control. (b) Quantification of single cell populations based on fluorescence profiles. Populations are classified as: intact (PI^–^/DiBAC^–^), depolarised (PI^–^/DiBAC^+^), depolarised and permeabilised (PI^+^/DiBAC^+^), and permeabilised (PI^+^/DiBAC^–^). Error bars represent mean ± SD from at least three biological replicates.

EGCG alone increased membrane permeability without significantly affecting polarisation. When combined with 760  kHz ultrasound, both permeabilisation and depolarisation were markedly intensified, indicating a frequency- and treatment-dependent membrane disruption pattern (Fig. 7). The higher permeabilisation effect observed at 760  kHz may be attributed to more efficient cavitation or mechanical stress acting on the phospholipid bilayer. Meanwhile, EGCG’s ability to increase membrane permeability could stem from its interaction with lipid domains and targeting outer membrane proteins, potentially weakening membrane packing [78]. When combined, EGCG may destabilise the membrane, making it more susceptible to ultrasound-induced stress, thus explaining the synergistic effect. Furthermore, increased membrane permeabilisation under ultrasound enhanced cellular uptake of EGCG, thereby strengthening the observed synergistic effect. This is consistent with previous studies demonstrating similar synergy. For example, ultrasound (20 kHz, 70% power for 9 min) combined with citral nanoemulsion disrupted *S. aureus* 29,213 cell membrane integrity, increased permeability, and elevated oxidative stress, as evidenced by FC, nucleic acid leakage assays, and SEM imaging [28].

**Fig. 8:**
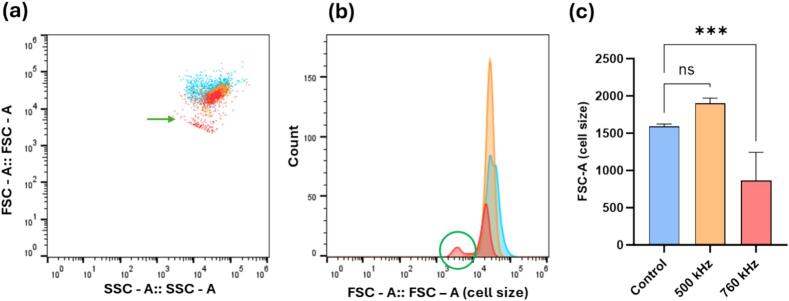
Flow cytometry analysis of *E. coli* cell size following different treatments. (a) FSC-A vs SSC-A scatter plot indicating single-cell populations; the green arrow highlights the reduced single-cell size cluster. (b) Density plot of FSC-A signal showing a leftward shift for treated cells (green circle), consistent with cell shrinkage and destruction. (c) Mean FSC-A intensity values quantified for each group. Error bars represent standard deviation from three replicates. Statistically significant differences are denoted as ***p < 0.001.

Exposure to 760 kHz treatment resulted in a pronounced decrease in single-cell size, evidenced by a distinct shift towards lower FSC-A values on FC plots (Fig. 8a-b) and a significant reduction in mean FSC-A compared to both control and 500 kHz treatment (*p* < 0.001, Fig. 8c). This trend was further supported by SEM-based bacterial length measurements (50 cells per condition) performed using ImageJ (Fig. S9). These results indicate HFUS results in cell shrinkage and/or cell destruction, likely through enhanced membrane permeability and associated water and/or ion fluxes, leading to decreased cell volume and structural compaction. Similar ultrasound‑induced physical disruption, including membrane damage, surface collapse and fluxes of intracellular contents (K^+^ and Ca^2+^), has been reported for *E. coli* O157:H7 exposed to high‑intensity low‑frequency ultrasound (20 kHz), where cells exhibited both mechanical injury and apoptosis‑like features [79]. Such morphological changes are a characteristic of stress-induced damage and may impact downstream physiological functions such as metabolism and motility [80]. Li et al. [81] proposed an “all-or-nothing” inactivation mechanism under LFUS (20  kHz), where cells were either fully inactivated or largely unaffected, based on double-staining FC in *E. coli* and *S. aureus*. By contrast, our results using DiBAC and PI under sublethal HFUS conditions revealed a more nuanced, “graded-injury” pattern. Cellular responses varied, with some cells showing mild damage and others more severe effects such as mild, moderate and severe membrane damage occurring in subpopulations, suggesting a heterogeneous response rather than a binary outcome. Notably, a decrease in FSC-A and SSC-A signals following HFUS exposure at 760 kHz (Fig. 8a) indicated reductions in cell size and internal complexity, respectively, as commonly interpreted in FC analyses [82]. These changes were likely due to mechanical stress, causing some cells to destruct and become more permeable while others remained relatively intact. The concentration of active bubble distribution near the liquid surface at 760 kHz, as evidenced by SL/SCL imaging (Fig. S2), may partly account for this graded-injury pattern, suggesting that not all cells were exposed to equivalent acoustic energy during treatment. This interpretation is consistent with previous reports showing that low‑intensity megasonic fields can differentially alter membrane permeability, with some mammalian keratinocyte cells displaying transient sonoporation and enhanced uptake whereas neighbouring cells maintain their basal state [83]. These morphological changes that are consistent with mechanical stress-induced shrinkage and destruction, were also observed in SEM images (Fig. 6d), reinforcing the evidence of ultrastructural alterations. Thus, FC not only confirmed membrane damage by HFUS but also highlighted morphological alterations that support the presence of a graded, rather than uniform, injury profile.

The graded injury profile observed under HFUS treatment may also be explained by bubble dynamics and bubble-cell interactions. HFUS typically generates smaller and near-resonant bubbles compared to those formed at LFUS. Brotchie et al. [84] estimated bubble resonance radii of 150, 14, 5.8, and 3.4 μm at 20, 213, 515, and 875 kHz, respectively, whereas *E. coli* cells have a radius of approximately 0.5 μm [85]. Using the acoustic wavelength relationship (c=λxν), the resonance bubble radii in the present system were estimated to be 6 μm at 500 kHz and ∼ 3.9 μm at 760 kHz, consistent with previously reported values. At HFUS, the resulting bubble-to-cell size ratio is therefore approximately 8- to 12-fold at 760 and 500 kHz, respectively. A decreased bubble-to-cell size ratio results in lower cell wall deformation, since the fraction of the bacterial surface under significant stress decreases [65]. Moreover, the percentage of damaged cells decreases steeply with increasing bubble–cell distance, following an approximate inverse cubic (1/r^3^) relationship. This indicates a highly localised interaction in which only cells passing very close to actively oscillating bubbles experience sufficient stress for membrane disruption [86]. Cells that undergo destruction during ultrasound exposure may become more susceptible to subsequent bubble-induced stresses, whereas cells that are not exposed to intense cavitation activity may remain intact. The combined influence of bubble-cell size ratio and bubble–cell distance therefore provides a plausible explanation for the graded injury patterns observed under HFUS, in which heterogeneous cellular responses arise within the same population (Fig. 8).

Having established that HFUS exerts both oxidative and physical effects on bacterial cells, a critical question arises as to whether sublethal oxidative exposure in particular may activate adaptive stress responses with implications for AMR.

### 3.5 Ultrasound-induced tolerance to lethal thermal and oxidative stress

**Fig. 9:**
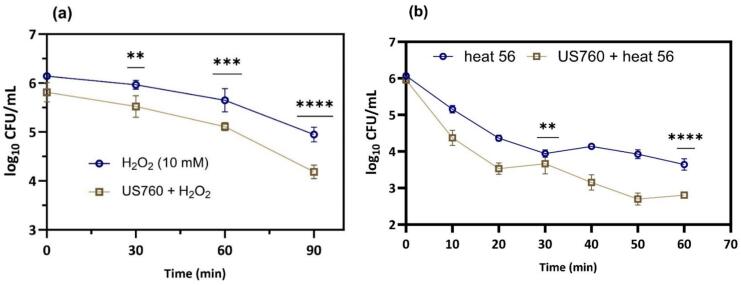
*E. coli* inactivation over time following (a) treatment with 10  mM H_2_O_2_ alone and in combination with prior ultrasonic exposure at 760  kHz, and (b) heat treatment at 56 °C alone and following prior ultrasonic pre-treatment. Error bars represent standard deviations from three independent replicates. ** *p* < 0.01, *** *p* < 0.001, **** *p* < 0.0001.

The potential for AMR development following non-thermal treatments has raised increasing concern, primarily because these interventions often exert mild and sublethal effects [32]. To determine if HFUS elicits stress responses that could contribute to AMR, bacterial samples were first exposed to 760 kHz at 30 W and subsequently challenged with lethal heat and oxidative stresses. HFUS-pretreated cells displayed increased sensitivity to both stresses compared with untreated controls (Fig. 9). This observation is consistent with the known effects of ultrasonic cavitation, where localised high temperatures, pressures, and free radical formation disrupt cellular membranes and sensitize bacteria to subsequent thermal damage [14]. In support of this, ultrasonic-assisted thermal treatment (thermosonication) has been shown to outperform single treatments against vegetative cells, spores and within broth and food matrices such as orange juice [14], [60].

Notably, pre-treatment with ultrasound prior to lethal oxidative stress exposure did not enhance bacterial survival but instead contributed to greater inactivation. This outcome suggests a synergistic interaction between ultrasound and H_2_O_2_. Ultrasound-induced cavitation increases membrane permeability, while H_2_O_2_ generates ROS that further damage cellular components [87]. For instance, Yadav et al. [88] reported strong synergistic effects when combining LFUS with H_2_O_2_ (2.1 mM/L) or ozonation concurrently in wastewater treatment, achieving synergy coefficients of 2.0 and 1.9, respectively, against a mixture of *Enterobacter*, *Salmonella*, and *E. coli*. These findings reinforce the notion that ultrasound can potentiate oxidative treatments rather than confer protection.

On the other hand, ultrasound can also impose multiple sublethal stresses simultaneously, including mild heating (approximately 42 °C), oxidative damage (e.g., 0.4 mM H_2_O_2_), and mechanical disruption at 760 kHz (30 W and 30 min). Bacteria subjected to such pre-ultrasound conditions exhibited altered survival profiles when subsequently challenged with lethal stressors (Fig. 9). In many cases, tolerance was reduced, most likely due to the cumulative burden of sublethal injury in this study. This outcome contrasts with classical stress-adaptation responses reported in the literature. For instance, Lou & Yousef [89] reported that *L. monocytogenes* exposed either to a non-lethal heat shock (45 °C, for 1 h) or to sublethal H_2_O_2_ (500 ppm, 1 h) exhibited significantly enhanced survival when subsequently challenged with lethal H_2_O_2_. Comparable findings in *E. coli* show that pre-heating at sublethal temperatures (45 °C for 30 or 5 min, depending on strain) can enhance thermotolerance during later lethal treatments [36]. Collectively, these studies highlight that sublethal stress can either sensitize or protect bacterial cells, depending on the nature and intensity of the stress encountered. Nevertheless, the greater inactivation observed in our study suggests that the combination of sublethal stresses induced by ultrasound, namely mild heat, oxidative, and mechanical effects, enhanced bacterial susceptibility to subsequent treatments. This aligns with previous reports showing that combined exposure to heat and oxidative stress can act synergistically, amplifying cellular stress responses and influencing survival outcomes [90]. Hence, the cumulative effect of such stresses may represent an important factor contributing to the improved efficacy of lethal applications [34].

Therefore, the current findings suggest that HFUS pre-treatment does not promote resistance development under tested conditions. Instead, by imposing simultaneous mechanical, thermal, and oxidative stresses, HFUS appears to compromise bacterial defence mechanisms and increase susceptibility to subsequent lethal challenges. This interpretation aligns with the observations in Sections 3.3 and 3.4, where HFUS was shown to disrupt membrane integrity and enhance oxidative damage, both of which may weaken bacterial resilience at the cellular level.

### 3.6 Stress response in HFUS sensitivity at the molecular level

**Fig. 10:**
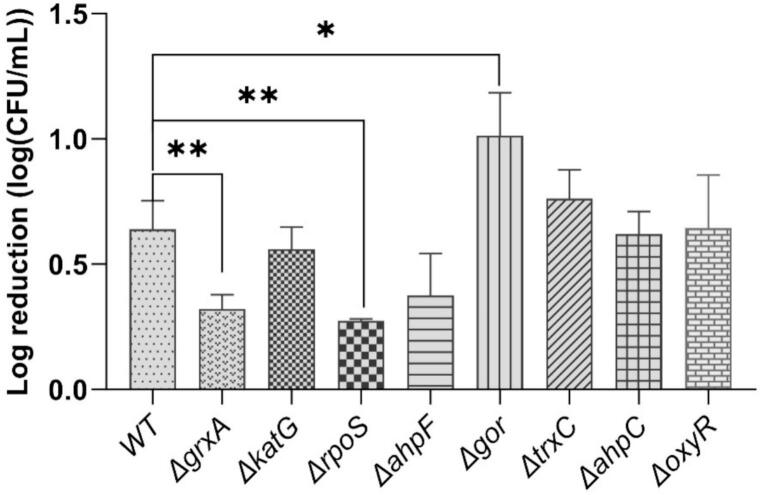
*E. coli* BW25113 wild-type (WT) and isogenic deletion mutants exposed to ultrasound at 760  kHz (30  W, 30  min, 1.7 cm). Bars represent the average log reduction in viable counts (CFU/mL). Error bars indicate standard deviation (n ≥ 3 independent repeats). Significant differences between WT and each mutant were determined by Student's *t*-test, with *p < 0.05 and **p < 0.01.

Although no enhanced tolerance to lethal treatments was observed under the tested conditions, previous studies have shown that ultrasonic exposure can modulate bacterial stress responses at the molecular level [31], [91], [92]. To investigate whether specific stress-response pathways contribute to HFUS sensitivity, eight *E. coli* K12 KEIO mutants and wild-type strain (BW25113), were exposed to 760 kHz ultrasound (30 W and 1.7 cm) for 30 min (Fig. 10).

Δ*gor*, a mutant in glutathione reductase, resulted in significantly increased sensitivity to ultrasound compared with the wild-type (*p* < 0.05). This heightened susceptibility is consistent with a weakened ability to maintain redox homeostasis and regenerate reduced glutathione, which may compromise cellular defense against HFUS-induced oxidative stress. However, because intracellular GSH/GSSG levels were not directly measured in this study, the extent of the contribution of glutathione imbalance could not be determined. Therefore, these results should be interpreted as suggesting a possible role for impaired glutathione recycling in HFUS sensitivity, rather than providing direct mechanistic proof [83].

In contrast, deletion of *grxA* (glutaredoxin 1) resulted in increased resistance to ultrasound. This response likely reflects the redundancy of the thiol-redox network in *E. coli*, whereby other redox proteins, such as thioredoxins (TrxA and TrxC), can compensate for the loss of GrxA and maintain redox balance under stress [93]. A similar increase in tolerance was observed for the Δ*rpoS* mutant. Although RpoS typically regulates the general stress response, and its deletion normally results in stress sensitivity we observed the opposite. Under certain conditions *rpoS* deletion can lead to compensatory regulatory shifts. In the context of HFUS exposure, altered baseline gene expression in Δ*rpoS* cells may activate alternative protective pathways, resulting in enhanced tolerance [92], [94]. Interestingly, similarly it has been demonstrated that the removal of the central stress gene regulator *sigB* in the Gram-positive bacterium *L. monocytogenes*, also results in an increased oxidative stress resistance [95]. These contrasting phenotypes highlight the non-redundant role of *gor* in antioxidant defence, while emphasising the stress response network flexibility *E. coli*’s that can buffer the loss of *grxA* or *rpoS* [94]*.*

## 4 Conclusions

The antimicrobial effects of HFUS were investigated using *E. coli* as a model organism to examine frequency-dependent antibacterial responses and associated stress mechanisms. Comparison of 500 and 760 kHz HFUS demonstrated the importance of system characterisation for optimising calorimetric power transmission, sonochemical activity, and antibacterial efficacy. Under the conditions tested, 760 kHz generated higher calorimetric power and sonochemical activity and resulted in greater bacterial inactivation than 500 kHz, demonstrating distinct antibacterial responses between the two HFUS frequencies investigated.

HFUS treatment was associated with increased intracellular ROS accumulation, membrane permeabilisation, changes in cell morphology, and enhanced leakage of intracellular contents. Flow cytometry and SEM analyses further indicated substantial structural stress and membrane damage following treatment. In addition, HFUS increased bacterial susceptibility to subsequent lethal exposures and enhanced the antibacterial activity of sublethal EGCG treatment. The increased sensitivity of the Δ*gor* mutant further suggests that oxidative stress responses may contribute to bacterial survival during HFUS exposure.

Collectively, the findings indicate that bacterial inactivation during HFUS treatment likely arises from the combined action of sonochemical, mechanical, and thermal effects associated with acoustic cavitation, and current evidence does not support attribution to any single dominant mechanism. While ROS generation and oxidative stress appear to contribute to the observed antibacterial effects, membrane disruption and other cavitation-related phenomena are also likely to play important roles. These results highlight the potential of HFUS as a non-thermal antimicrobial technology and as a tool to enhance the efficacy of complementary antimicrobial interventions.

Future studies should investigate the relative contributions of extracellular and intracellular oxidative processes, membrane-associated effects, and cavitation phenomena, while extending evaluation to a broader range of HFUS frequencies, additional foodborne pathogens, and complex food matrices.

**Data Availability Statement.**

The data that support the findings of this study are available from the corresponding author upon reasonable request.

**AI Use Declaration**

During the preparation of this work, Irem Soyler used Claude (Anthropic) to check grammar and improve flow. Following the use of this tool, the author reviewed and edited the content as necessary and takes full responsibility for the content of the published article.

## CRediT authorship contribution statement

**Irem Soyler:** Writing – review & editing, Writing – original draft, Visualization, Validation, Project administration, Methodology, Investigation, Formal analysis, Data curation, Conceptualization. **Katie Costello-Gould:** Writing – review & editing, Supervision, Resources, Conceptualization. **Kimon Andreas G. Karatzas:** Writing – review & editing, Resources, Methodology. **Jorge Gutierrez-Merino:** Writing – review & editing, Validation, Supervision, Resources, Project administration, Methodology, Conceptualization. **Madeleine Bussemaker:** Writing – review & editing, Supervision, Resources, Project administration, Methodology, Funding acquisition, Formal analysis, Conceptualization.

## Declaration of competing interest

The authors declare that they have no known competing financial interests or personal relationships that could have appeared to influence the work reported in this paper.

## Appendix A Supplementary data

Supplementary Data 1 Supplementary figures (Figs. S1-S9) and tables (Tables S1-S2) containing supporting information for the experimental methods and results described in the main manuscript.

## References

[1] L. Astráin-Redín, M. Alejandre, J. Raso, G. Cebrián, and I. Álvarez, “Direct contact ultrasound in food processing: impact on food quality,” Jan. 28, 2021, *Frontiers Media S.A.* doi: 10.3389/fnut.2021.633070. 10.3389/fnut.2021.633070 PMC7876345 33585542

[2] Ashokkumar M. (2011). “The characterization of acoustic cavitation bubbles - an overview,” in *Ultrasonics Sonochemistry*. Elsevier b.v..

[3] Millan-Sango D., McElhatton A., Valdramidis V.P. (Jan. 2015). Determination of the efficacy of ultrasound in combination with essential oil of oregano for the decontamination of *Escherichia coli* on inoculated lettuce leaves. Food Res. Int..

[4] H. B. Jadhav, U. S. Annapure, and R. R. Deshmukh, “Non-thermal Technologies for Food Processing,” Jun. 08, 2021, *Frontiers Media S.A.* doi: 10.3389/fnut.2021.657090. 10.3389/fnut.2021.657090 PMC8217760 34169087

[5] Zhang H., Tsai S., Tikekar R.V. (Mar. 2021). Inactivation of Listeria innocua on blueberries by novel ultrasound washing processes and their impact on quality during storage. Food Control.

[6] G. Zheng, Z. Tang, and F. Peng, “Ultrasound-activated inorganic nanomaterials to generate ROS for antibacterial applications,” Apr. 10, 2025, *Royal Society of Chemistry*. doi: 10.1039/d5bm00121h. 10.1039/d5bm00121h 40241671

[7] Costello K.M. (Nov. 2021). The effect of ultrasound treatment in combination with nisin on the inactivation of Listeria innocua and *Escherichia coli*. Ultrason. Sonochem..

[8] Zhang H. (2020). Inactivation of foodborne pathogens based on synergistic effects of ultrasound and natural compounds during fresh produce washing. Ultrason. Sonochem..

[9] J. Dai, M. Bai, C. Li, H. Cui, and L. Lin, “Advances in the mechanism of different antibacterial strategies based on ultrasound technique for controlling bacterial contamination in food industry,” Nov. 01, 2020, *Elsevier Ltd*. doi: 10.1016/j.tifs.2020.09.016.

[10] B. Duan, X. Shao, Y. Han, Y. Li, and Y. Zhao, “Mechanism and application of ultrasound-enhanced bacteriostasis,” Mar. 25, 2021, *Elsevier Ltd*. doi: 10.1016/j.jclepro.2020.125750.

[11] K. S. Suslick, “Acoustic Cavitation in Homogeneous Liquids,” *Science (1979).*, vol. 247, no. 4949, pp. 1439–1445, 1990, [Online]. Available: https://www.science.org.

[12] S. I. Nikitenko, “Plasma formation during acoustic cavitation: Toward a new paradigm for sonochemistry,” 2014, *Hindawi Publishing Corporation*. doi: 10.1155/2014/173878.

[13] Huang J., Fu Q., Shao X., Li Y. (Jul. 2025). Ultrasonic strategies for mitigating microbial adhesion and biofilm formation on medical surfaces: a mini review. Front. Microbiol..

[14] Yasasvi Rathnayake P., Na Yu R., Eun Yeo S., Choi Y.-S., In Yong H. (2025). Application of Ultrasound to Animal-based Food to Improve Microbial Safety and Processing Efficiency 2 3 running Title: Ultrasound applications for Microbial Safety in Animal Foods. Food Sci. Anim. Resour..

[15] Mason T.J., Cobley A.J., Graves J.E., Morgan D. (2011). New evidence for the inverse dependence of mechanical and chemical effects on the frequency of ultrasound. Ultrason. Sonochem..

[16] Gao S., Lewis G.D., Ashokkumar M., Hemar Y. (2014). Inactivation of microorganisms by low-frequency high-power ultrasound: 1. effect of growth phase and capsule properties of the bacteria. Ultrason. Sonochem..

[17] A. Thi Hong Bui, D. Cozzolino, B. Zisu, and J. Chandrapala, “Effects of high and low frequency ultrasound on the production of volatile compounds in milk and milk products - A review,” Nov. 01, 2020, *Cambridge University Press*. doi: 10.1017/S0022029920001107. 10.1017/S0022029920001107 33353571

[18] Y. Cai, J. Wang, X. Liu, R. Wang, and L. Xia, “A review of the combination therapy of low frequency ultrasound with antibiotics,” 2017, *Hindawi Limited*. doi: 10.1155/2017/2317846. 10.1155/2017/2317846 PMC5662814 29124063

[19] Nahum Y., Cerrone A., Nerenberg R. (Mar. 2025). Impact of Low-Frequency Ultrasound on Physical Properties and Antibiotic Susceptibility of a Mucoid Biofilm. Langmuir.

[20] M. S. Granick, C. Paribathan, M. Shanmugam, and N. Ramasubbu, “Direct-Contact Low-Frequency Ultrasound Clearance of Biofilm From Metallic Implant Materials,” 2017. PMC5372756 28405263

[21] G. Matafonova and V. Batoev, “Review on low- and high-frequency sonolytic, sonophotolytic and sonophotochemical processes for inactivating pathogenic microorganisms in aqueous media,” Dec. 01, 2019, *Elsevier Ltd*. doi: 10.1016/j.watres.2019.115085. 10.1016/j.watres.2019.115085 31539667

[22] Wu X., Yan L., Xu G., Wang X., Wang J.J., Dionysiou D.D. (Nov. 2021). High frequency ultrasonication enhances iron-catalyzed sulphate inactivation of *Escherichia coli* and *Staphylococcus aureus*. Chemical Engineering Journal Advances.

[23] Gao S., Hemar Y., Ashokkumar M., Paturel S., Lewis G.D. (Sep. 2014). Inactivation of bacteria and yeast using high-frequency ultrasound treatment. Water Res..

[24] S. N. Guerrero, M. Ferrario, M. Schenk, and M. G. Carrillo, “Hurdle Technology Using Ultrasound for Food Preservation,” in *Ultrasound: Advances in Food Processing and Preservation*, Elsevier Inc., 2017, pp. 39–99. doi: 10.1016/B978-0-12-804581-7.00003-8.

[25] S. C. Forester and J. D. Lambert, “The role of antioxidant versus pro-oxidant effects of green tea polyphenols in cancer prevention,” Jun. 2011. doi: 10.1002/mnfr.201000641. 10.1002/mnfr.201000641 PMC3679539 21538850

[26] M. Wu and A. C. Brown, “Applications of catechins in the treatment of bacterial infections,” *Pathogens*, vol. 10, no. 5, 2021, doi: 10.3390/pathogens10050546. 10.3390/pathogens10050546 PMC8147231 34062722

[27] Takundwa B.A., Bhagwat P., Ruzengwe F.M., Pillai S., Ijabadeniyi O.A. (2022). Optimisation of the combined treatment of nisin, oregano and ultrasound in decontaminating *Listeria monocytogenes* and *Escherichia coli* O157:H7 on cabbage. Future Foods.

[28] Yang H. (Apr. 2023). Synergistic antibacterial and anti-biofilm mechanisms of ultrasound combined with citral nanoemulsion against *Staphylococcus aureus* 29213. Int. J. Food Microbiol..

[29] Zhang L., Qi H., Yan Z., Gu Y., Sun W., Zewde A.A. (Jan. 2017). Sonophotocatalytic inactivation of E. coli using ZnO nanofluids and its mechanism. Ultrason. Sonochem..

[30] M. Nikoo, J. M. Regenstein, and H. Ahmadi Gavlighi, “Antioxidant and Antimicrobial Activities of (-)-Epigallocatechin-3-gallate (EGCG) and its Potential to Preserve the Quality and Safety of Foods,” May 01, 2018, *Blackwell Publishing Inc.* doi: 10.1111/1541-4337.12346. 10.1111/1541-4337.12346 33350134

[31] Spiteri D., Chot-Plassot C., Sclear J., Karatzas K.A., Scerri C., Valdramidis V.P. (Oct. 2017). Ultrasound processing of liquid system(s) and its antimicrobial mechanism of action. Lett. Appl. Microbiol..

[32] Velliou E.G., Van Derlinden E., Cappuyns A.M., Geeraerd A.H., Devlieghere F., Van Impe J.F. (Jan. 2012). Heat inactivation of *Escherichia coli* K12 MG1655: effect of microbial metabolites and acids in spent medium. J. Therm. Biol.

[33] S. Bouillet, T. S. Bauer, and S. Gottesman, “RpoS and the bacterial general stress response,” *Microbiology and Molecular Biology Reviews*, vol. 88, no. 1, Mar. 2024, doi: 10.1128/mmbr.00151-22. 10.1128/mmbr.00151-22 PMC10966952 38411096

[34] M. Boura, M. M. Yilmaz Topcam, D. Spiteri, C. Bruschi, V. Valdramidis, and K. A. G. Karatzas, “Acid or salt adaptation of *Listeria monocytogenes* 10403S grown until exponential phase aerobically, enhances sensitivity to oxidative stress,” *J. Appl. Microbiol.*, vol. 136, no. 7, Jul. 2025, doi: 10.1093/jambio/lxaf173. 10.1093/jambio/lxaf173 40657895

[35] S. Patil, P. Bourke, and B. Cullen, “The Effects of Acid Adaptation on Escherichia Coli Inactivation Using Power Ultrasound,” 2009. [Online]. Available: https://arrow.tudublin.ie/schfsehart.

[36] Li R., Shi Y., Ling B., Cheng T., Huang Z., Wang S. (2017). Thermo-tolerance and heat shock protein of *Escherichia coli* ATCC 25922 under thermal stress using test cell method. Emir. J. Food Agric..

[37] Li Y., Lv R., Zhou J., Wang W., Liu D. (2024). Efficacy and antibacterial mechanism of high-frequency ultrasound combined with sodium hypochlorite against E. coli O157:H7. J. Food Process Eng.

[38] Baba T. (2006). Construction of *Escherichia coli* K-12 in-frame, single-gene knockout mutants: the Keio collection. Mol. Syst. Biol..

[39] Fujishiro K., Okada S., Rossi F., Kuchimaru T., Kurashina Y. (May 2026). Intracellular Reactive Oxygen Species Generation Induced by High-Frequency Ultrasound in Thickness Vibration Mode. Adv. Sci..

[40] Koda S., Kimura T., Kondo T., Mitome H. (2003). A standard method to calibrate sonochemical efficiency of an individual reaction system. Ultrason. Sonochem..

[41] Zare M. (Mar. 2023). A fundamental study on the degradation of paracetamol under single- and dual-frequency ultrasound. Ultrason. Sonochem..

[42] Atkinson E.R., Lawler H.J., Heath J.C., Kimball E.H., Awtrey A.D., Connick R.E. (1946). “The Bleaching. Dyeing and Chemical Technology of Textile Fibers”.

[43] Hart E.J., Henglein A. (1985). “Free Radical and Free Atom Reactions in the Sonolysis of Aqueous. Iodide and Formate Solutions”.

[44] Rivas D. (Nov. 2012). Sonoluminescence and sonochemiluminescence from a microreactor. Ultrason. Sonochem..

[45] Ashokkumar M. (2010). Spatial distribution of acoustic cavitation bubbles at different ultrasound frequencies. ChemPhysChem.

[46] Dolan H.L., Bastarrachea L.J., Tikekar R.V. (2018). Inactivation of Listeria innocua by a combined treatment of low-frequency ultrasound and zinc oxide. LWT.

[47] Alfonso-Prieto M., Biarnés X., Vidossich P., Rovira C. (Aug. 2009). The molecular mechanism of the catalase reaction. J. Am. Chem. Soc..

[48] H. Kim and X. Xue, “Detection of Total Reactive Oxygen Species in Adherent Cells by 2’,7’-Dichlorodihydrofluorescein Diacetate Staining,” 2021. 10.3791/60682 PMC7712457 32658187

[49] Nikolaou A. (Mar. 2025). The ratio of reactive oxygen and nitrogen species determines the type of cell death that bacteria undergo. Microbiol. Res..

[50] Moghimi R., Ghaderi L., Rafati H., Aliahmadi A., Mcclements D.J. (Mar. 2016). Superior antibacterial activity of nanoemulsion of Thymus daenensis essential oil against E. coli. Food Chem..

[51] Chen Y.Y., Gänzle M.G. (Apr. 2016). Influence of cyclopropane fatty acids on heat, high pressure, acid and oxidative resistance in *Escherichia coli*. Int. J. Food Microbiol..

[52] Uhl L., Gerstel A., Chabalier M., Dukan S. (2015). Hydrogen peroxide induced cell death: one or two modes of action?. Heliyon.

[53] A. T. Mustapha, H. Wahia, Q. Ji, O. A. Fakayode, L. Zhang, and C. Zhou, “Multiple-frequency ultrasound for the inactivation of microorganisms on food: A review,” Apr. 01, 2024, *John Wiley and Sons Inc*. doi: 10.1111/jfpe.14587.

[54] Hua I., Thompson J.E. (Oct. 2000). Inactivation of *Escherichia coli* by sonication at discrete ultrasonic frequencies. Water Res..

[55] Zhu Y., Zhu X., Pan X., Liu L.X., Bussemaker M.J. (2025). Correlation of sonochemical activities measured via dosimetry and an area-selective analysis of sono(chemi)luminescence. RSC Mechanochemistry.

[56] Beckett M.A., Hua I. (Apr. 2001). Impact of ultrasonic frequency on aqueous sonoluminescence and sonochemistry. J. Phys. Chem. A.

[57] Wood R.J., Lee J., Bussemaker M.J. (Sep. 2017). A parametric review of sonochemistry: Control and augmentation of sonochemical activity in aqueous solutions. Ultrason. Sonochem..

[58] Nikitenko S.I., Le Naour C., Moisy P. (2007). Comparative study of sonochemical reactors with different geometry using thermal and chemical probes. Ultrason. Sonochem..

[59] Aguilar C., Serna-Jiménez J., Benitez E., Valencia V., Ochoa O., Sotelo L.I. (Apr. 2021). Influence of high power ultrasound on natural microflora, pathogen and lactic acid bacteria in a raw meat emulsion. Ultrason. Sonochem..

[60] Tian P., Wang X., Zhu G., Zhang H., Zhang Z. (2025). Effects of combining lysozyme, ultrasound, and heat treatments on the inactivation of *Bacillus subtilis* spores. Arch. Microbiol..

[61] Gomez-Gomez A., Brito-de la Fuente E., Gallegos C., Garcia-Perez J.V., Benedito J. (Aug. 2021). Combination of supercritical CO2 and high-power ultrasound for the inactivation of fungal and bacterial spores in lipid emulsions. Ultrason. Sonochem..

[62] Noci F., Walkling-Ribeiro M., Cronin D.A., Morgan D.J., Lyng J.G. (Jan. 2009). Effect of thermosonication, pulsed electric field and their combination on inactivation of Listeria innocua in milk. Int. Dairy J..

[63] Pflieger R., Chave T., Vite G., Jouve L., Nikitenko S.I. (Sep. 2015). Effect of operational conditions on sonoluminescence and kinetics of H2O2 formation during the sonolysis of water in the presence of Ar/O2 gas mixture. Ultrason. Sonochem..

[64] Giuntini F. (Jun. 2018). Insight into ultrasound-mediated reactive oxygen species generation by various metal-porphyrin complexes. Free Radic. Biol. Med..

[65] Zinin P.V., Allen J.S. (2007). P4E-5 Theoretical Analysis of Oscillations of Cells in the High Frequency Ultrasonic Field. IEEE Ultrason. Symp..

[66] M. J. Moreno-Vásquez *et al.*, “Characterization of epigallocatechin-gallate-grafted chitosan nanoparticles and evaluation of their antibacterial and antioxidant potential,” *Polymers (Basel).*, vol. 13, no. 9, May 2021, doi: 10.3390/polym13091375. 10.3390/polym13091375 PMC8122830 33922410

[67] J. Steinmann, J. Buer, T. Pietschmann, and E. Steinmann, “Anti-infective properties of epigallocatechin-3-gallate (EGCG), a component of green tea,” Mar. 2013. doi: 10.1111/bph.12009. 10.1111/bph.12009 PMC3594666 23072320

[68] S. D. Falcinelli, M. C. Shi, A. M. Friedlander, and J. Chua, “Green tea and epigallocatechin-3-gallate are bactericidal against *Bacillus anthracis*,” *FEMS Microbiol. Lett.*, vol. 364, no. 12, Jul. 2017, doi: 10.1093/femsle/fnx127. 10.1093/femsle/fnx127 28605495

[69] Yoda Y., Hu Z.Q., Zhao W.H., Shimamura T. (2004). Different susceptibilities of Staphylococcus and Gram-negative rods to epigallocatechin gallate. J. Infect. Chemother..

[70] Ma S., Shi C., Wang C., Guo M. (Oct. 2017). Effects of ultrasound treatment on physiochemical properties and antimicrobial activities of whey protein-totarol nanoparticles. J. Food Prot..

[71] Zhang S. (Sep. 2022). Ultrasound-assisted preparation of lactoferrin-EGCG conjugates and their application in forming and stabilizing algae oil emulsions. Ultrason. Sonochem..

[72] Zheng Y., Chen B., Huang X., Teng H., Ai C., Chen L. (May 2023). Ultrasound-assisted free radical modification on the structural and functional properties of ovalbumin-epigallocatechin gallate (EGCG) conjugates. Ultrason. Sonochem..

[73] Liu Z. (Mar. 2025). Synergistic inactivation effect of ultrasound and nano-emulsified basil essential oil on the metabolic responses of Salmonella on sprouts. Int. J. Food Microbiol..

[74] J. D. Lambert and R. J. Elias, “The antioxidant and pro-oxidant activities of green tea polyphenols: A role in cancer prevention,” Sep. 2010. doi: 10.1016/j.abb.2010.06.013. 10.1016/j.abb.2010.06.013 PMC2946098 20558130

[75] Xiong L.G. (Feb. 2017). Tea polyphenol epigallocatechin gallate inhibits *Escherichia coli* by increasing endogenous oxidative stress. Food Chem..

[76] Cui Y. (2012). AFM probing the Mechanism of Synergistic Effects of the Green Tea Polyphenol (-)-Epigallocatechin-3-Gallate (EGCG) with Cefotaxime against Extended-Spectrum Beta-Lactamase (ESBL)-Producing *Escherichia coli*. PLoS One.

[77] Li X. (Nov. 2025). Ultrasound cavitation modulates intracellular ROS and gene expression in antibacterial sonodynamic therapy. Ultrason. Sonochem..

[78] Nakayama M. (Oct. 2013). A study of the antibacterial mechanism of catechins: Isolation and identification of *Escherichia coli* cell surface proteins that interact with epigallocatechin gallate. Food Control.

[79] J. Li *et al.*, “Ultrasound-induced *Escherichia coli* O157:H7 cell death exhibits physical disruption and biochemical apoptosis,” *Front. Microbiol.*, vol. 9, no. OCT, Oct. 2018, doi: 10.3389/fmicb.2018.02486. 10.3389/fmicb.2018.02486 PMC6232819 30459727

[80] F. Lang *et al.*, “Functional Significance of Cell Volume Regulatory Mechanisms,” 1998. 10.1152/physrev.1998.78.1.247 9457175

[81] Li J., Ahn J., Liu D., Chen S., Ye X., Ding T. (2016). Evaluation of ultrasoundinduced damage to *Escherichia coli* and *Staphylococcus aureus* by flow cytometry and transmission electron microscopy. Appl. Environ. Microbiol..

[82] J. Śliwa-Dominiak *et al.*, “Flow Cytometry in Microbiology: A Review of the Current State in Microbiome Research, Probiotics, and Industrial Manufacturing,” Mar. 01, 2025, *John Wiley and Sons Inc*. doi: 10.1002/cyto.a.24920. 10.1002/cyto.a.24920 40028773

[83] Domenici F. (2017). Differential effects on membrane permeability and viability of human keratinocyte cells undergoing very low intensity megasonic fields. Sci. Rep..

[84] A. Brotchie, F. Grieser, and M. Ashokkumar, “Effect of power and frequency on bubble-size distributions in acoustic cavitation,” *Phys. Rev. Lett.*, vol. 102, no. 8, Feb. 2009, doi: 10.1103/PhysRevLett.102.084302. 10.1103/PhysRevLett.102.084302 19257742

[85] M. Riley, “Correlates of Smallest Sizes for Microorganisms,” in *Size Limits of Very Small Microorganisms: Proceedings of a Workshop*, Washington , 1999.

[86] J. Wu, “• Original Contribution THEORETICAL STUDY ON SHEAR STRESS GENERATED BY MICROSTREAMING SURROUNDING CONTRAST AGENTS ATTACHED TO LIVING CELLS,” 2002. 10.1016/s0301-5629(01)00497-5 11879959

[87] Zhu Y. (Dec. 2022). The synergistic antibacterial activity and mechanism of ultrasound and hydrogen peroxide against *Staphylococcus aureus* in water. J. Water Process Eng..

[88] Yadav M., Gole V.L., Sharma J., Yadav R.K. (Dec. 2022). Biologically treated industrial wastewater disinfection using the synergy of low-frequency ultrasound and H2O2/O3. J. Environ. Health Sci. Eng..

[89] Lou Y., Yousef A.E. (1997). “Adaptation to Sublethal Environmental Stresses Protects *Listeria monocytogenes* against Lethal Preservation. Factors”.

[90] Crombie T.A., Tang L., Choe K.P., Julian D. (Jul. 2016). Inhibition of the oxidative stress response by heat stress in Caenorhabditis elegans. J. Exp. Biol..

[91] Li J., Zhang X., Ashokkumar M., Liu D., Ding T. (Mar. 2020). Molecular regulatory mechanisms of *Escherichia coli* O157:H7 in response to ultrasonic stress revealed by proteomic analysis. Ultrason. Sonochem..

[92] Spiteri D., Griffin S., Karatzas K.A., Scerri C., Valdramidis V.P. (2023). *Escherichia coli* K-12 Transcriptomics for Assessing the Mechanism of Action of High-Power Ultrasound. Microorganisms.

[93] L. R. Knoke, M. Muskietorz, L. Kühn, and L. I. Leichert, “Comprehensive elucidation of glutathione import in *Escherichia coli*,” Jul. 16, 2024. doi: 10.1101/2024.07.15.603537.

[94] Gayán E., Wang Z., Salvador M., Gänzle M.G., Aertsen A. (Feb. 2023). Dynamics of high hydrostatic pressure resistance development in RpoS-deficient *Escherichia coli*. Food Res. Int..

[95] Boura M. (2016). Loss of sigb in listeria monocytogenes strains egd-e and 10403s confers hyperresistance to hydrogen peroxide in stationary phase under aerobic conditions. Appl. Environ. Microbiol..

