# Supplementary material for: Investigating antimicrobial stress responses in Escherichia coli induced by high-frequency ultrasound
Source: Ultrason Sonochem. 2026 Jun 27;132:107935. doi: 10.1016/j.ultsonch.2026.107935 (PMC13380529; doi:10.1016/j.ultsonch.2026.107935)
Supplement: Supplementary Data 1 — Supplementary figures (Figs. S1-S9) and tables (Tables S1-S2) containing supporting information for the experimental methods and results described in the main manuscript. [file mmc1.docx]

**Investigating antimicrobial stress responses in​ *Escherichia coli* induced by high-frequency ultrasound**

Irem Soyler^a^, Katie Costello-Gould^b^, Kimon-Andreas Karatzas^c^, Jorge Gutierrez-Merino^d^, Madeleine Bussemaker^a^

^a^ School of Chemistry and Chemical Engineering, Department of Chemical and Process Engineering, University of Surrey, Guildford, United Kingdom

^b^ Fluor Limited*, Farnborough, United Kingdom

^c^ School of Chemistry, Food & Pharmacy, Department of Food and Nutritional Sciences, University of Reading, United Kingdom

^d^ School of Veterinary Medicine, Faculty of Health and Medical Sciences, University of Surrey, Guildford, United Kingdom

* *This document was developed with the permission of Fluor as part of the Fluor P4 Program. Note that the views expressed in this document may be directed to a large audience and may not be suitable for any particular client, project, or situation. Fluor does not provide any warranties relating to the content of this document and your use or reliance on it is at your own risk. © 2026 Fluor Corporation. All rights reserved. Fluor is a registered service mark of Fluor Corporation.*

**
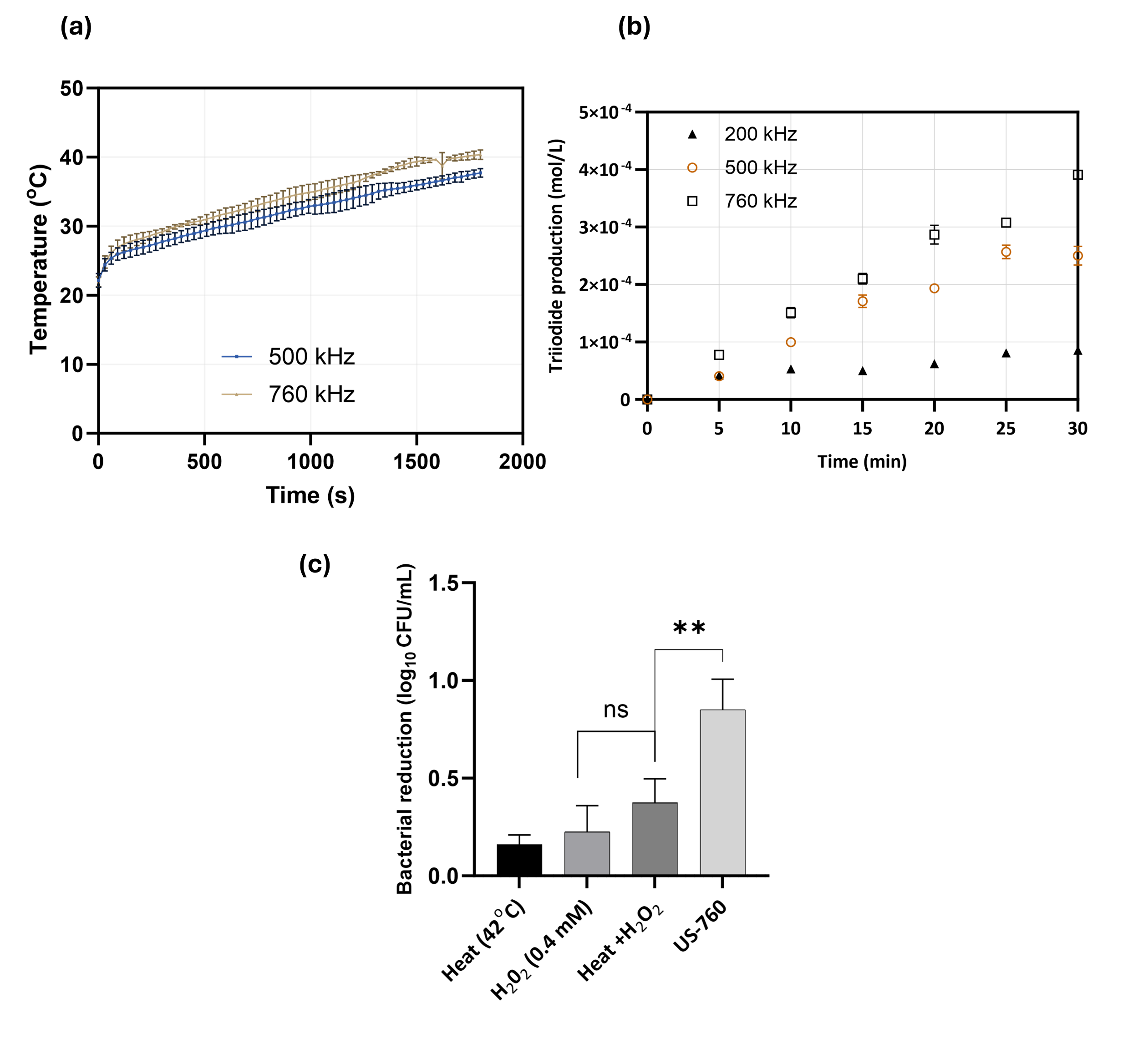
Supplementary Materials**

**Figure S1.** Preliminary characterisation of ultrasonic treatment conditions and non-ultrasonic thermal and chemical controls. (a) Bulk temperature profiles recorded at 500 and 760 kHz (30 W) over 30 min, measured every 30 s. (b) *E. coli* inactivation following exposure to temperature, hydrogen peroxide, and their combination at levels corresponding to the maximum conditions generated during 760 kHz HFUS treatment (30 W, 30 min), in the absence of ultrasound. (c) Sonochemical activity assessed by KI dosimetry at 200, 500, and 760 kHz under identical operating conditions (30 W, 30 min).

Figure S2 presents sonoluminescence (SL) and sonochemiluminescence (SCL) images acquired at 500 and 760 kHz using water (SL) and luminol solution (SCL) at volumes of 350, 500, and 750 mL, and serves to supplement the spatial observations shown in Figure 3 of the main manuscript. Figure S3 provides the corresponding quantitative analysis of SL and SCL intensities across the tested frequencies and liquid volumes.


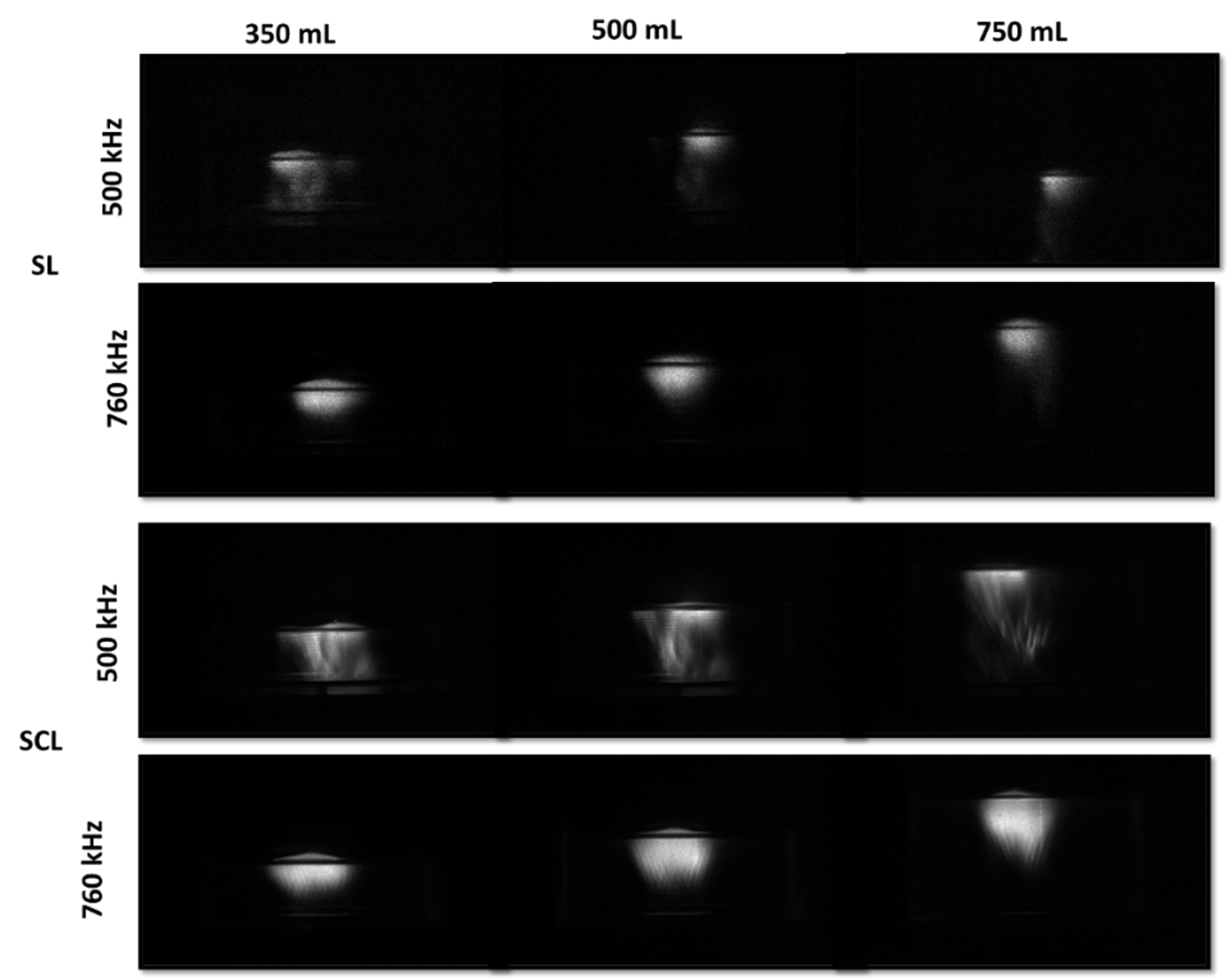


**Figure S2**. Sonoluminescence (SL) and sonochemiluminescence (SCL) images recorded at 500 and 760 kHz using water (SL) and luminol solution (SCL) at volumes of 350, 500, and 750 mL.


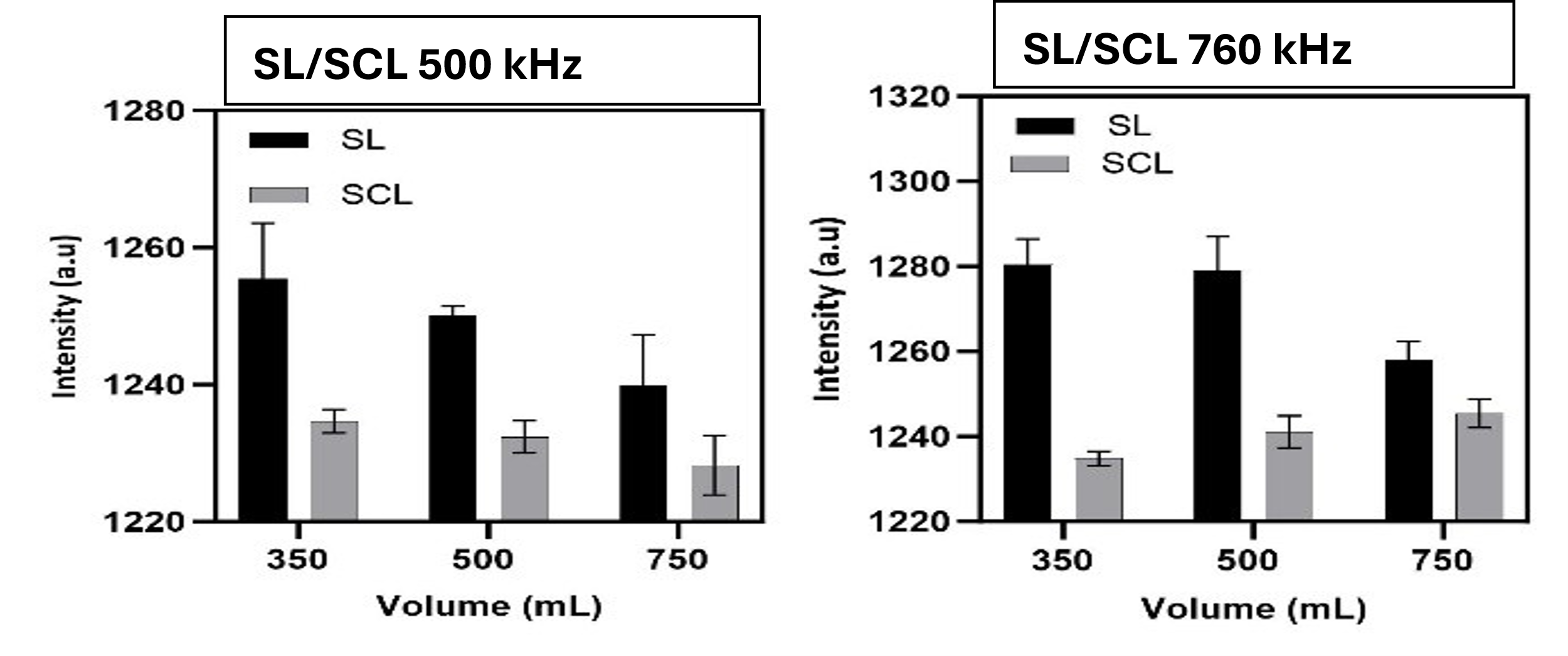


**Figure S3.** Quantification of SL and SCL intensity corresponding to the SL–SCL images shown in Figure S1 at 500 and 760 kHz across varying liquid volumes.

The concentration of L-histidine used for the radical scavenger assay was determined using KI dosimetry. Figure S4 shows the formation of triiodide ions (I₃⁻) following ultrasonic treatment at 760 kHz and 30 W for 10 min, quantified spectrophotometrically. Figure S5 presents representative images of the KI solutions, illustrating the corresponding yellow colour development associated with triiodide formation.

**Figure S4.** Triiodide (I₃⁻) production at 760 kHz and 30 W for 10 min in the presence of varying concentrations of L-histidine.


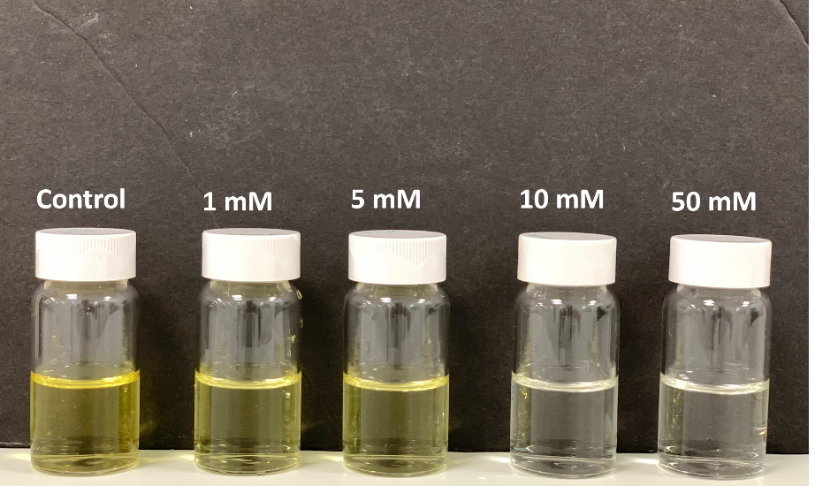


**Figure S5.** Visual illustration of triiodide (I₃⁻) formation at different L-histidine concentrations following ultrasonic treatment at 760 kHz and 30 W for 10 min.

Figure S6 illustrates the assessment of epigallocatechin gallate (EGCG) degradation and ROS generation during HFUS treatment. A calibration curve was established, and ultrasonically treated samples (760 kHz, 30 W, 30 min) were analysed to quantify residual EGCG concentration, allowing evaluation of ultrasound-induced degradation. To investigate the contribution of sonochemically generated ROS, KI dosimetry was performed in the presence of 0.2 mg/mL EGCG during ultrasonic treatment (760 kHz, 30 W, 10 min). The results demonstrated the generation of oxidative species under ultrasonic conditions and provided insight into the interaction between EGCG and sonochemically produced radicals. Furthermore, intracellular ROS production in *Escherichia coli* was assessed by flow cytometry using the DCF assay following treatment with ultrasound alone and ultrasound combined with EGCG (760 kHz, 30 W, 30 min). Quantification of DCF-positive cells revealed increased intracellular oxidative stress following ultrasonic treatment, supporting the involvement of ROS in the antimicrobial activity observed under these conditions. Collectively, these findings provide mechanistic evidence supporting the combined HFUS-EGCG treatment and serve as important control experiments to evaluate the interaction of EGCG with sonochemically generated ROS, including its potential antioxidant activity, thereby complementing the results presented in the main manuscript.


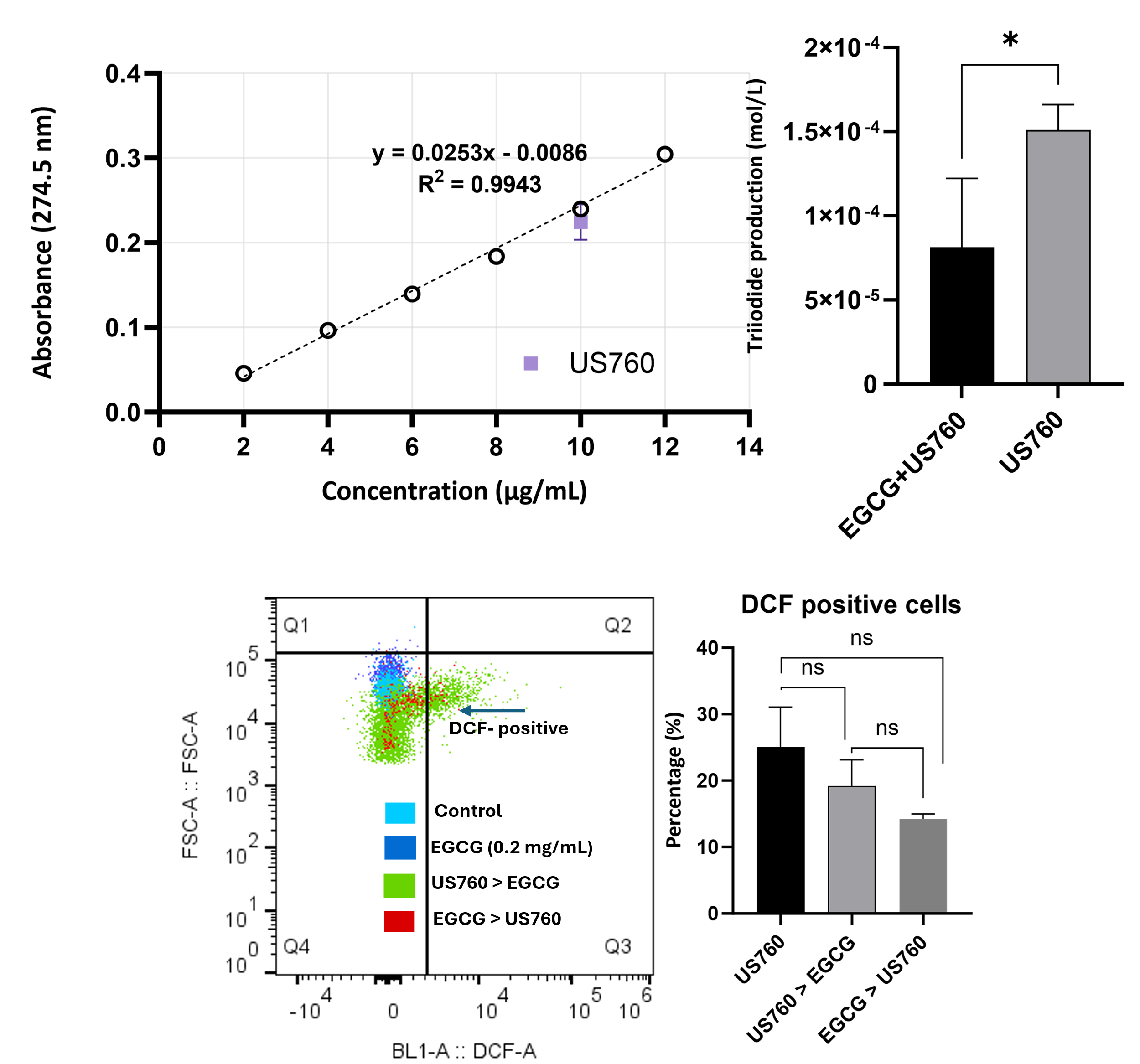


**Figure S6.** Assessment of EGCG degradation and reactive oxygen species (ROS) generation during high-frequency ultrasound treatment. Top-left: calibration curve used for EGCG quantification and determination of EGCG degradation following ultrasonic treatment at 760 kHz and 30 W for 30 min. Top-right: potassium iodide (KI) dosimetry in the presence of 0.2 mg/mL EGCG during ultrasonic treatment at 760 kHz and 30 W for 10 min, indicating sonochemically generated oxidants. Bottom: flow cytometric quantification of DCF-positive *Escherichia coli* cells following treatment with ultrasound alone (760 kHz, 30 W, 30 min) and ultrasound combined with EGCG, indicating intracellular ROS accumulation.


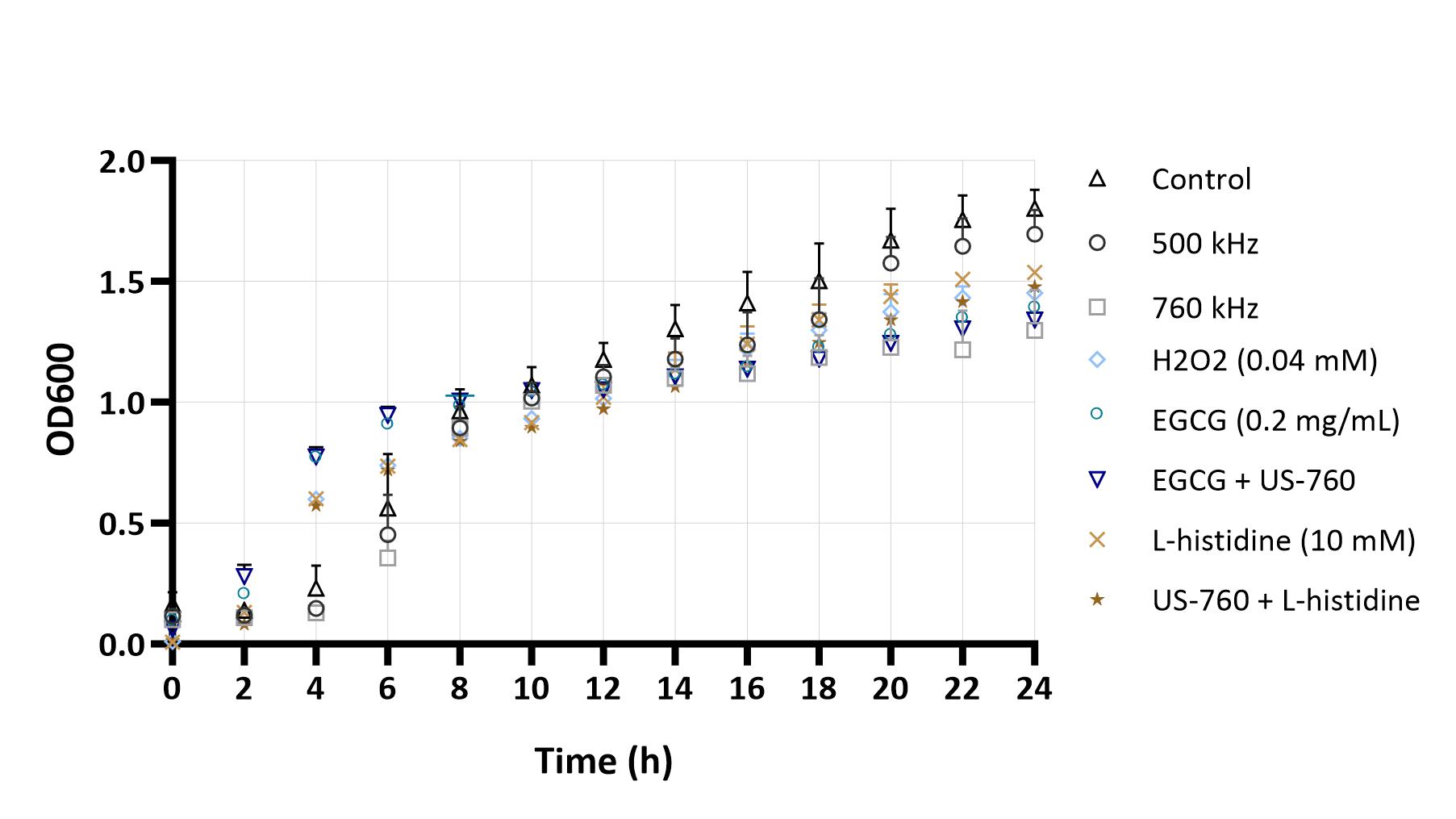
Figure S7 is provided to supplement Figure 4c in the main manuscript and presents OD600 measurements after 24 h of regrowth for ultrasonically treated samples compared with untreated controls. Additional treatment conditions were also included for completeness but are not discussed in detail in the main text.

**Figure S7.** OD600 measurements of *E. coli* after 24 h of regrowth following different treatments.

Figure S8 presents SEM images of EGCG-treated samples in the absence of bacteria, provided to supplement Figure 7c in the main manuscript. These images demonstrate that EGCG residues do not accumulate on surfaces in the absence of bacterial cells, regardless of ultrasonic treatment at 760 kHz and 30 W for 30 min. Any residues observed in the images are therefore likely attributable to artefacts introduced during SEM sample preparation rather than to EGCG deposition.


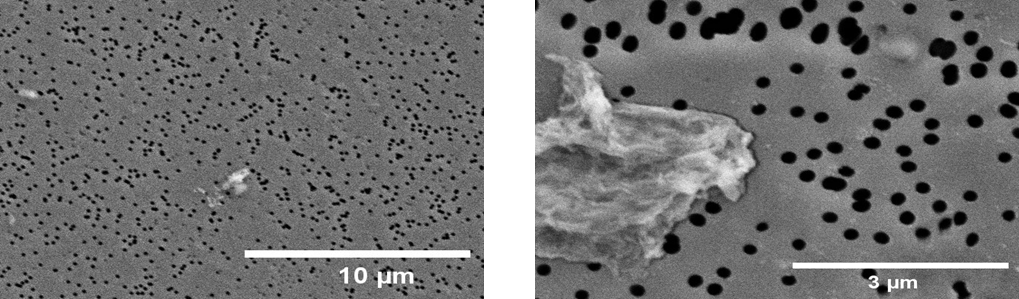


**Figure S8.** SEM images of EGCG samples in the absence of bacteria following ultrasonic treatment at 760 kHz and 30 W for 30 min.

Figure S9 shows bacterial length measurements obtained from SEM images for control, 500 kHz, and 760 kHz treatments using ImageJ. For each condition, approximately 50 cells were measured across 6–7 images to minimise bias, and all clearly measurable cells were included in the analysis.


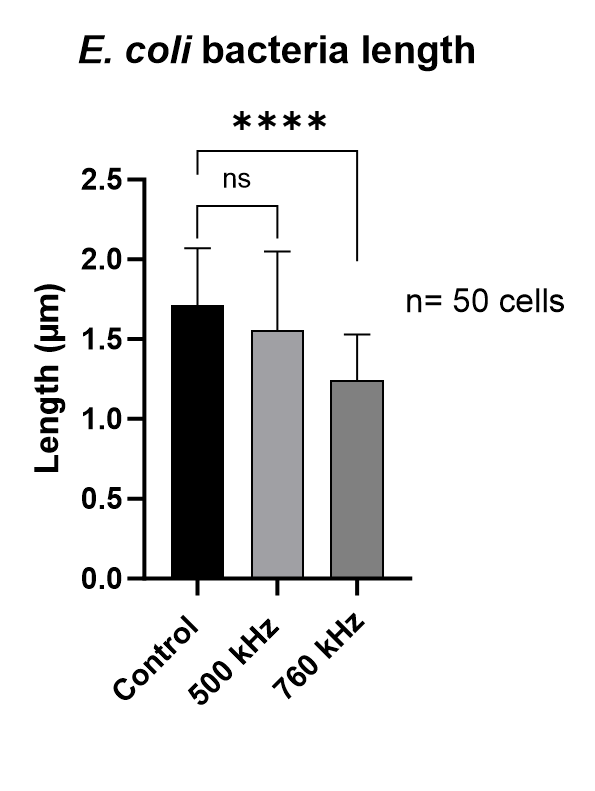


**Figure S9.** SEM-based bacterial cell length measurements by Image J. Error bars represent standard deviation (SD). Statistical significance is indicated as **** (*p* < 0.0001) and ns (not significant).

**Table S1.** Two-way ANOVA results evaluating the effects of ultrasonic frequency and sample positioning, and their interaction, on triiodide production.

| ANOVA - Triiodide Production (mol/L) | | | | | |
| --- | --- | --- | --- | --- | --- |
|  | **Sum of Squares** | **df** | **Mean Square** | **F** | **p** |
| **Height (cm)** | 1.18e-8 | 2 | 5.92e-9 | 17.0 | <.001 |
| **Frequency (kHz)** | 2.39e-7 | 1 | 2.39e-7 | 685.1 | <.001 |
| **Height (cm) ✻ Frequency (kHz)** | 5.22e-8 | 2 | 2.61e-8 | 74.8 | <.001 |
| **Residuals** | 1.67e-8 | 48 | 3.49e-10 |  |  |

**Table S2.** Post hoc multiple comparison analysis of triiodide (I₃⁻) production across experimental conditions.

| Post Hoc Comparisons - Height (cm) ✻ Frequency (kHz) | | | | | | | | | |
| --- | --- | --- | --- | --- | --- | --- | --- | --- | --- |
| **Comparison** | | | | |  | | | | |
| **Height (cm)** | **Frequency (kHz)** |  | **Height (cm)** | **Frequency (kHz)** | **Mean Difference** | **SE** | **df** | **t** | **p_tukey_** |
| **1.0** | **500** | **-** | **1.0** | **760** | -5.13e−5 | 8.80e-6 | 48.0 | -5.83 | <.001 |
|  |  | **-** | **1.7** | **500** | 2.06e-5 | 8.80e-6 | 48.0 | 2.34 | 0.200 |
|  |  | **-** | **1.7** | **760** | -1.25e−4 | 8.80e-6 | 48.0 | -14.22 | <.001 |
|  |  | **-** | **2.0** | **500** | 4.07e-5 | 8.80e-6 | 48.0 | 4.62 | <.001 |
|  |  | **-** | **2.0** | **760** | -1.61e−4 | 8.80e-6 | 48.0 | -18.33 | <.001 |
|  | **760** | **-** | **1.7** | **500** | 7.19e-5 | 8.80e-6 | 48.0 | 8.17 | <.001 |
|  |  | **-** | **1.7** | **760** | -7.38e−5 | 8.80e-6 | 48.0 | -8.38 | <.001 |
|  |  | **-** | **2.0** | **500** | 9.20e-5 | 8.80e-6 | 48.0 | 10.45 | <.001 |
|  |  | **-** | **2.0** | **760** | -1.10e−4 | 8.80e-6 | 48.0 | -12.50 | <.001 |
| **1.7** | **500** | **-** | **1.7** | **760** | -1.46e−4 | 8.80e-6 | 48.0 | -16.55 | <.001 |
|  |  | **-** | **2.0** | **500** | 2.01e-5 | 8.80e-6 | 48.0 | 2.29 | 0.220 |
|  |  | **-** | **2.0** | **760** | -1.82e−4 | 8.80e-6 | 48.0 | -20.67 | <.001 |
|  | **760** | **-** | **2.0** | **500** | 1.66e-4 | 8.80e-6 | 48.0 | 18.84 | <.001 |
|  |  | **-** | **2.0** | **760** | -3.62e−5 | 8.80e-6 | 48.0 | -4.12 | 0.002 |
| **2.0** | **500** | **-** | **2.0** | **760** | -2.02e−4 | 8.80e-6 | 48.0 | -22.95 | <.001 |
| Note. Comparisons are based on estimated marginal means | | | | | | | | | |
